# Soluble PD-L1 as an early marker of progressive disease on nivolumab

**DOI:** 10.1136/jitc-2021-003527

**Published:** 2022-02-07

**Authors:** Kathleen M Mahoney, Petra Ross-Macdonald, Long Yuan, Linan Song, Eliseo Veras, Megan Wind-Rotolo, David F McDermott, F Stephen Hodi, Toni K Choueiri, Gordon J Freeman

**Affiliations:** 1 Department of Medical Oncology, Dana-Farber Cancer Institute, Boston, Massachusetts, USA; 2 Department of Medicine, Beth Israel Deaconess Medical Center, Boston, Massachusetts, USA; 3 Bristol-Myers Squibb Co, Princeton, New Jersey, USA; 4 Department of Immunology, Harvard Medical School, Boston, Massachusetts, USA; 5 Quanterix Corporation, Billerica, Massachusetts, USA

**Keywords:** melanoma, biomarkers, tumor, immunoassay, immunotherapy, kidney neoplasms

## Abstract

**Background:**

Soluble PD-L1 (sPD-L1) has been associated with worse prognosis in numerous solid tumors. We determined sPD-L1 levels before and during nivolumab treatment in two prospective clinical trials of metastatic clear cell renal cell carcinoma (RCC) and melanoma patients, and investigated its relationship to clinical factors, biomarkers, and outcome.

**Methods:**

Using a new Single Molecule Array assay, serum sPD-L1 level were determined in RCC (CheckMate 009, n=91) and melanoma (CheckMate 038-Part 1, n=78) prior to, and at two time points on treatment. Gene expression data was obtained from biopsies taken prior to, and at day 28 on treatment. Results were integrated with clinical variables, tumor PD-L1 status from immuno-histochemistry, and genomic mutation status.

**Results:**

In RCC patients, sPD-L1 levels were higher in patients with progressive disease as their best response. For both RCC and melanoma patients, progressive or stable disease was associated with an increase in sPD-L1 on nivolumab therapy, whereas mean sPD-L1 levels did not change or declined in patients with objective responses. By categorizing RCC patients into transcriptomic molecular subtypes, we identified a subgroup where the associations between sPD-L1 and progressive disease were particularly evident. In baseline biopsies, we identified six biological processes that were associated with sPD-L1 level in both RCC and melanoma: higher sPD-L1 is associated with lower tumor expression of the Hallmark gene sets ‘hypoxia’, ‘fatty acid metabolism’, ‘glycolysis’, ‘MTORC1 signaling’ and ‘androgen response’, and with higher expression of ‘KRAS signaling_Down’.

**Conclusion:**

Baseline and on-therapy sPD-L1 levels in RCC have the potential to predict progressive disease on PD-1 inhibitor nivolumab. In a hypothesis-generating analysis of tumor gene expression, high baseline sPD-L1 is associated with a tumor metabolic state reflecting potentially targetable processes in both melanoma and RCC. In both trials, we observed associations between change in sPD-L1 on treatment and outcome metrics. sPD-L1 levels may further refine a nivolumab-refractory subtype of RCC within transcriptionally based subtypes of RCC.

## Background

An ideal biomarker would be accessible in the periphery, relevant across multiple tumor types, and provide information to guide initial selection, combination and sequencing of therapies. Regulatory approvals for the PD-1 family of therapeutics have outpaced the development of clinically useful biomarkers for their use. Finding biomarkers for immunotherapies has proven to be particularly challenging, in part due to the dynamic nature of the tumor microenvironment and its relationship to the tumor. While sampling tumors for PD-L1 expression, tumor mutational burden (TMB) and inflammation have identified associations with response to PD-1 blockade therapy in multiple cancers including melanoma, renal cell carcinoma (RCC) has remained an outlier with respect to their utility.^1^ RCC is consistently and highly inflamed but has a lower TMB than most types of PD-1 responsive tumors.^2^ For RCC, transcription-based tumor subtypes have proved to be indicators of response, for both tyrosine kinase inhibitors (TKIs) and immunotherapies.^3 4^ A common biomarker for these tumors is lacking.

### PD-L1 in the tumor

The PD-1 ligand (PD-L1) is a coinhibitory molecule expressed on immune cells (T cells, B cells, dendritic cells, and macrophages) as well as some non-hematologic cells, such as the placenta. Tumor cells can also express PD-L1 through oncogene activation or induction by interferon-gamma.^5^ PD-L1-positivity of tumor cells and/or immune cells in the tumor microenvironment by immunohistochemistry (IHC) was the earliest biomarker to show association with response to PD-1 blockade.^6^ However, intratumoral PD-L1 protein expression has not proven useful as a biomarker in most settings for clinical triage because of its limited positive and negative predictive value, due in part to the absence of standardization across antibodies, tumors and immune checkpoint blockers as well as the importance of PD-L1 in the lymph node in some tumors.^6–8^ In kidney cancer, tumor PD-L1 is a well-established marker of poor prognosis and is associated with worse outcomes in RCC patients treated with TKIs.^9 10^ Tumor PD-L1 expression was associated with higher response rates to PD-1 blockade in melanoma and non-small cell lung cancer (NSCLC) patients, but there was no significant difference in advanced RCC.^11^

### Soluble PD-L1

Since PD-L1 may be expressed dynamically by both tumor cells and immune cells in the tumor microenvironment, soluble PD-L1 (sPD-L1) may be a more accessible and relevant surrogate for the total expression of PD-L1 by tumors (reviewed in Ref. 13). PD-L1 is a transmembrane protein and primarily expressed on the surface of cells, but multiple forms of sPD-L1 have been described: cleaved, secreted splice variants and exosomal PD-L1.^12^ Culture supernatants of PD-L1- expressing tumor cell lines and activated dendritic cells contain high levels of sPD-L1.^9 13^ A meta-analysis of sPD-L1 in different solid tumors (n=1040) that included multiple studies with RCC, found that high levels of soluble PD-L1 (sPD-L1) in peripheral blood were associated with poor prognosis.^14^ While for some solid tumors such as NSCLC, sPD-L1 is elevated in patients with >50% tumor cell positivity for PD-L1, this relationship was not seen in RCC.^12^ In a retrospective analysis of melanoma patients, those with high sPD-L1 in the peripheral blood prior to treatment developed progressive disease (PD) on CTLA4 blockade. The majority of patients also had an increase in sPD-L1 after therapy, suggesting sPD-L1 may be a pharmacodynamic marker for CTLA4 blockade.^15^

There is a clear need for liquid biomarkers that are broadly relevant to immunotherapy. We have developed an ultrasensitive capture assay to detect sPD-L1 using the Quanterix Single Molecule Array (SIMOA) technology. In this study, we quantified sPD-L1 in prospectively collected serum samples from patients in two biomarker-directed clinical trials of PD-1 monotherapy: advanced RCC (CheckMate 009) and melanoma (CheckMate 038 part 1). Using sPD-L1 levels at baseline and two on-treatment timepoints, we could also determine the dynamics within each patient. Somatic mutation status and gene expression data from pretreatment and on-treatment biopsies were available for a subset of patients in both trials. This study identifies clinical and biological factors associated with sPD-L1 levels and provides a comprehensive evaluation of the association between sPD-L1 metrics and therapeutic outcomes. We find that sPD-L1 is a dynamic marker of early PD on nivolumab in patients with either kidney cancer or melanoma.

## Methods

### Study design

CheckMate 009 (NCT01358721) was an open-label, parallel, four-group, phase 1b study of nivolumab in advanced renal cancer (Bristol Myers Squibb; Ono Pharmaceutical Company Limited). Part 1 of CheckMate 038 (CheckMate 038-P1; NCT01621490) was an open-label, single arm, two group phase 1 study of nivolumab in advanced melanoma (Bristol Myers Squibb). Study design, methods, and baseline clinical and demographic features have been previously described.^16 17^ Serum on CheckMate 009 was obtained at baseline, at day 29 and day 63. Serum on CheckMate 038 was obtained at baseline, at day 29 and day 43. In both trials, paired fresh biopsies from metastatic lesions were obtained at baseline and study day 29. All patients gave written informed consent.

### Response metrics

Tumor burden change (maximum reduction or minimum increase in index lesions) and Best Overall Response (BOR) were determined by Response Evaluation Criteria in Solid Tumors V.1.1. For analyses that require a binary definition of response (ie, responder or non-responder), response status was defined to concur with previously published studies for CheckMate 009^18 19^ or Objective Response (complete response (CR)+partial response (PR) vs stable disease (SD)+PD+ NE) for CheckMate 038-P1.

### SIMOA assay and serum analyses

**Figure 1:**
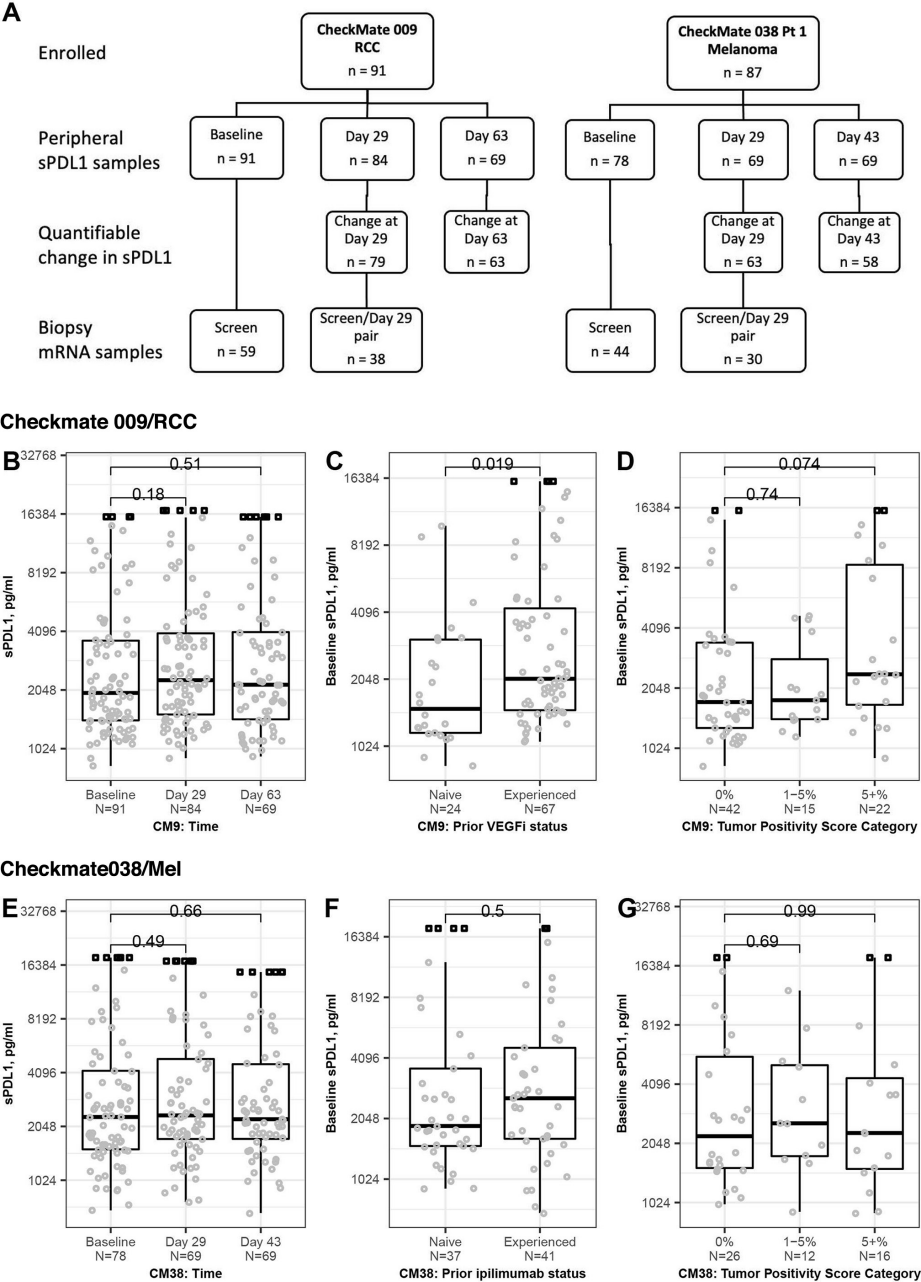
Sample collection and association of sPD-L1 with clinical characteristics. For association with clinical characteristics, measurable serum level of sPD-L1 in patients from CheckMate 009 (B–D) and CheckMate 038-P1 (E–G) are indicated by circles. For samples where sPD-L1 level exceeded the ULOQ, the imputed values are indicated by square symbols. All p values derived from Wilcoxon rank sum test. (A) Schematic of sPD-L1 and Affymetrix RNA datasets used in this analysis. Samples where sPD-L1 level exceeded the ULOQ were not used in analyses of change on treatment. (B) sPD-L1 level at baseline (n=91), day 29 (n=84) and day 63 (n=69) of nivolumab therapy in CheckMate 009. (C) sPD-L1 level at baseline (n=91) in CheckMate 009 by 1L patients (Naïve, n=24) or patients with >1 prior therapy including a VEGFi (Experienced, n=67). (D) sPD-L1 level at Baseline in CheckMate 009 in patients with PD-L1 scoring of their tumor biopsy. Data are grouped by percentage of biopsied tumor cells that stain positive for PD-L1: 0% (n=42), 1% to 5% (n=15) or over 5% (n=22). (E) sPD-L1 level at Baseline (n=78), day 29 (n=69) and day 43 (n=69) of nivolumab therapy in CheckMate 038-P1. (F) sPD-L1 level at Baseline (n=78) in CheckMate 038-P1. Data are grouped by patients who have not previously been treated with ipilimumab (Naïve, n=37) or patients with ipilimumab therapy at some point prior to enrollment (Experienced, n=41). (G) sPD-L1 level at baseline in CheckMate 038-P1 in patients with PD-L1 scoring of their tumor biopsy. Data are grouped by percentage of biopsied tumor cells that stain positive for PD-L1: 0% (n=26), 1% to 5% (n=12) or over 5% (n=16). RCC, renal cell carcinoma; ULOQ, upper limit of quantitation; VEGFi, VEGFR inhibitor.

A previous ELISA assay (anti-human PD-L1 mAb 29E.12B1/capture and biotinylated anti-PD-L1 mAb 29E.2A3 (Biolegend)/detector) had a lower limit of detection sensitivity of 100 pg/mL of recombinant PD-L1.^15^ Screening of multiple pairs of PD-L1 antibodies developed in the Freeman laboratory in a SIMOA^20 21^ assay (Quanterix, Billerica, MA) identified 29E.12B1 (capture) with 339.4C10 (detector) as the strongest pair, with a lower limit of detection sensitivity of 3 pg/mL of recombinant PD-L1 for this retrospective study. SIMOA assays were performed blinded to clinical treatments and outcomes on 100 µL of serum, obtained at baseline (prior to initiation of nivolumab) and at day 29 in both trials, and at day 43 for CheckMate 038-P1 or day 63 for CheckMate 009. sPD-L1 values are the average of two assays for each sample (online supplemental table S1). For 34 samples whose result exceeded the upper limit of quantitation (ULOQ) despite dilution, an imputed value was assigned based on the mean and 3.5-fold SD calculated on all samples in the trial at the assay time point. Imputed values were used in the time point-specific analyses, for example, figure 1, but are not included in analyses of change over time.

10.1136/jitc-2021-003527.supp1 Supplementary data

### Tumor analyses

Paired fresh frozen biopsies from metastatic lesions at baseline and study day 29 were used to evaluate tumor-associated lymphocytes, PD-L1 status, somatic genome sequence, and gene expression. IHC assessment of tumor-associated lymphocytes (Mosaic Laboratories, Lake Forest, California, USA) and PD-L1 expression on the tumor cell surface (Dako PD-L1 IHC 28–8 pharmDx) have been described previously.^16^

### Gene expression analyses

RNA was labeled by WT-Pico Ovation (NuGEN, San Carlos, California, USA) and profiled using the HG-U219 array on the GeneTitan platform (Affymetrix, Santa Clara, California, USA). Robust multi-array average expression values were determined for 18,562 loci (BrainArray V.10). Affymetrix data were evaluable for 72 patients in CheckMate 009 (n=59 at baseline, 55 at day 29, 42 matched) and 65 patients in CheckMate 038-P1 (n=49 at baseline, 56 at day 29, 40 matched). Gene signature scores were calculated for the ‘Angiogenesis’, ‘T-effector’ and ‘Myeloid Inflammation’ gene sets described in the IMmotion150 trial publication (5), the Tumor Inflammation Signature (33), and ‘cytolytic’ gene set,^22^ the 26 genes evaluated as previously described in JAVELIN Renal 101 trial (‘JAVELIN’) publication (11), and an EMT/stromal gene set associated with T-cell exclusion (36). Gene set scores were calculated as the median value of Z-scored expression level for the constituent transcripts. We also interrogated a set of 60 genes encoding metalloproteases (MMP/ADAM genes). For CheckMate 009 the ‘ccrcc’ subtype was assigned by WARD hierarchical clustering of baseline expression data for 63 available transcripts (from the 70-gene panel.^3^ This method recapitulated the ccrcc assignment in the original dataset E-MTAB-3267. All gene sets used are provided in online supplemental table S2. Estimates for the abundance of immune cell populations were derived using CIBERSORT.^23^ For analyses of correlation with immune infiltrate at baseline, we required at least 20% of samples had the cell type present. For analyses of correlation with change in immune infiltrate, we required the cell type to be present at one of the time points. For CheckMate 009, reliability of CIBERSORT estimates was confirmed by agreement with CD4 and CD8 scoring by IHC (data not shown). Analyses of the relationship of baseline gene expression to baseline sPD-L1 level used limma (Bioconductor V.3.8.^24^ For Gene Set Enrichment Analysis (GSEA), ranked results for all 18,562 genes were evaluated with the ‘GSEA’ algorithm (Bioconductor V.3.8;.^25^ ‘Hallmark’ curated gene sets were from MSigDb.^26^

### Statistical analyses

Association of discrete factors with response was evaluated using Fisher’s exact test. Hazard for survival was estimated from Cox proportional hazard models. HRs compare the highest tertile to the lowest tertile (tertile analyses), the above median group to the below median group (median-split analyses) or nominal patients whose sPDL1 values differ by IQR (continuous analyses). Association of discrete factors with sPD-L1 level was evaluated using the Kruskal-Wallis rank sum test, the receiver operating characteristic (ROC) and the area under curve (AUC). Association of numeric factors with sPD-L1 level was evaluated using Pearson, Spearman and Kendall correlation.

### Data and code availability

Gene expression data and annotation are in ArrayExpress (E-MTAB-3218, E-MTAB-4030). Analyses performed in R V.3.6.3 are available on github.com/rossmacp/sPDL1_ Publication).

## Results

### Generation of serum sPD-L1 biomarker data

This study uses samples collected prospectively in two biomarker-focused clinical trials of nivolumab monotherapy. CheckMate 009 enrolled first-line (1L) and vascular endothelial growth factor receptor (VEGFR) inhibitor (VEGFi)-experienced (2+L) patients with advanced RCC, while CheckMate 038-part 1 enrolled several types of advanced melanoma and included a cohort that had previously been treated with ipilimumab. Both studies specified characterization of serum cytokine levels and tumor immune infiltration as primary outcomes. To this end, samples of sera were obtained at multiple time points on treatment, and the data available from pretreatment and on-treatment tumor biopsies includes IHC for PD-L1, gene expression and somatic mutation status.

We applied our ultrasensitive SIMOA sPD-L1 assay (Methods) with sera samples obtained from patients enrolled in CheckMate 009/RCC (n=91) and CheckMate 038-P1 (n=78). sPD-L1 levels were determined at baseline (prior to initiation of nivolumab), at day 29, and at day 43 for CheckMate 038-P1 or Day 63 for CheckMate 009 (online supplemental table S1). Over 90% of the 465 samples had appropriate linearity in the assay; 7% (34) of samples were above the ULOQ (16 ng/mL).

### Association between baseline sPD-L1 and clinical characteristics

We evaluated association between baseline sPD-L1 and clinical characteristics of the patient populations (table 1). In CheckMate 009/RCC, we observed no significant association between sPD-L1 level and the time on therapy (figure 1A). We observed that baseline sPD-L1 was significantly lower in the naive cohort (n=24) relative to patients who had experienced one or more prior lines of therapy including a VEGFi (p=0.019; figure 1B). In RCC patients with ≥5% tumor cells staining positive for PD-L1, sPD-L1 level was generally higher (p=0.074), but otherwise, we saw no association between baseline sPD-L1 and the percentage of PD-L1-positive tumor cells in biopsies obtained at baseline (figure 1C). There was also no association with age (<65 years vs ≥65 years), sex or somatic mutation status for PBRM1 or VHL1 (online supplemental figure S1A–D).

10.1136/jitc-2021-003527.supp2 Supplementary data

**Table 1:** Median values of sPDL1 or change in sPDL1 (pg/mL) for patient cohorts in CM-009 and CM-038-P1

Trial	Cohort	Baseline (n=)	Baseline (pg/mL)	Day 29 (pg/mL)	Day 43 or 63 (pg/mL)	Baseline to day 29 (paired samples n=)	Baseline to day 29 (change in pg/mL)	Baseline to day 43 or 63 (paired samples, n=)	Baseline to day 43 or 63 (change in pg/mL)
RCC CM-009	Trial cohort	*91*	1978	2300	2179	*79*	180	*63*	114
	2+L cohort	*67*	2057	2374	2332	*57*	185	*42*	95
	PD	*27*	3138	3397	4043	*25*	259	*14*	224
	SD	*42*	1809	1999	1866	*38*	103	*35*	134
	CRPR	*14*	1635	2115	1692	*14*	42	*13*	−83
Melanoma CM-038	Trial cohort	*78*	2312	2361	2247	*63*	175	*58*	171
	PD	*34*	2183	2065	2238	*27*	175	*22*	243
	SD	*24*	2452	2654	2505	*21*	188	*19*	160
	CRPR	*20*	1936	1970	2133	*15*	79	*17*	70

Samples with imputed values are included in calculation of the time point-specific medians, but are not included in any analyses of change over time. The second time point is day 63 in CM-009 and day 43 in CM-038-P1.

CRPR, complete or partial response; PD, progressive disease; RCC, renal cell carcinoma; SD, stable disease.

In CheckMate 038-P1/melanoma, we observed no significant association between sPD-L1 level and the time on therapy (figure 1D). We saw no association of baseline sPD-L1 with experience of prior CTLA4 therapy (figure 1E) or with the percentage of PD-L1-positive tumor cells in biopsies obtained at baseline (figure 1F). There was no association of baseline sPD-L1 with age (<65 years vs ≥65 years) or sex (online supplemental figure S1E, F). The baseline sPD-L1 level was generally higher in patients with mucosal melanoma compared with patients with cutaneous melanoma, suggesting a difference in the biology of the subtypes. However, any conclusions would require a larger sample size, given there are few noncutaneous subtypes in this cohort (p=0.059 for 50 cutaneous vs 7 mucosal; online supplemental figure S1G). Baseline sPD-L1 was significantly higher in CheckMate 038-P1 patients whose LDH exceeded the normal upper limit (p=0.003; online supplemental figure S1H).

### Association between sPD-L1 level and clinical response

**Figure 2:**
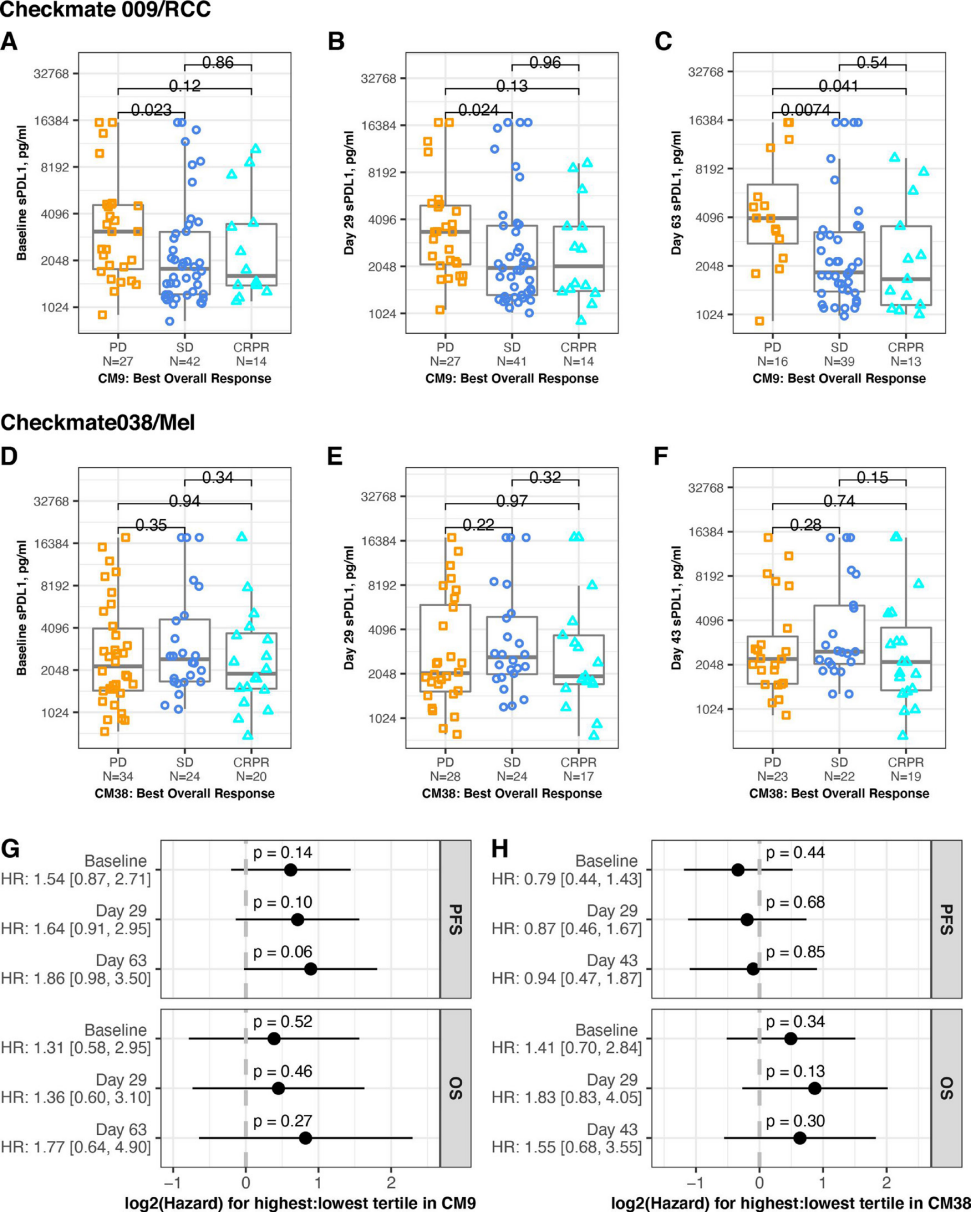
Association between sPD-L1 and response. Serum level of sPD-L1 in patients from CheckMate 009 (upper panels) and CheckMate038 (middle panels). BOR is indicated by gold squares (PD), blue circles (SD) or aqua triangles (CR or PR). All p values are derived from Wilcoxon rank sum test. (A) Samples provided at baseline in CheckMate 009. Data are grouped by BOR (PD, SD or CRPR, n=27, 42, 14, respectively). (B) Samples provided at day 29 in CheckMate 009. Data are grouped by BOR (PD, SD or CRPR, n=27, 41, 14 respectively). (C) Samples provided at day 63 in CheckMate 009. Data are grouped by BOR (PD, SD or CRPR, n=16, 39, 13, respectively). (D) Samples provided at baseline in CheckMate 038-P1. Data are grouped by BOR (PD, SD or CRPR, n=34, 24, 20, respectively). (E) Samples provided at day 29 in CheckMate 038-P1. Data are grouped by BOR (PD, SD or CRPR, n=28, 24, 17, respectively). (F) Samples provided at day 43 in CheckMate 038-P1. Data are grouped by BOR (PD, SD or CRPR, n=23, 22, 19, respectively). (G) Cox proportional hazard analysis of survival in CheckMate 009, comparing patients from the highest tertile of sPD-L1 values to patients from the lowest tertile of values, for each timepoint indicated. Panel displays p value and zero-centered HR. HR and 95% CIs are indicated to left. (H) Cox proportional hazard analysis of survival in CheckMate 038-P1, comparing patients from the highest tertile of sPD-L1 values to patients from the lowest tertile of values, for each timepoint indicated. Panel displays p value and zero-centered HR. HR and 95% CIs are indicated to left. BOR, best overall response; CR, complete response; OS, overall survival; PFS, progression-free survival; PD, progressive disease; PR, partial response; SD, stable disease.

We evaluated association between the sPD-L1 level at each timepoint and the patient’s BOR (PD, SD, CRPR). In CheckMate 009/RCC, median sPD-L1 was higher at all timepoints in patients with PD relative to SD or CRPR (table 1, figure 2A, B and C). At baseline and both on-treatment time points, there was significantly higher mean level of sPD-L1 in patients with PD compared with patients with SD. There was significantly higher mean level of sPD-L1 in patients with PD compared with patients with CR+PR at day 63. In CheckMate 038-P1/melanoma, there was no significant association between sPD-L1 and BOR at any timepoint (table 1, figure 2D, E and F). Using the binary response status, which combines SD +PD (see the Methods section), we found no association to levels of sPD-L1 at baseline or on treatment in either CheckMate 009/RCC or CheckMate 038-P1/melanoma (online supplemental figure S2A–F).

We also evaluated association between sPD-L1 level and progression-free survival (PFS) or overall survival (OS). Hazard for PFS was increased in the highest tertile in CheckMate 009/RCC (p=0.06 on treatment at day 63), but there was no statistically significant difference in hazard for PFS or OS at any time point in either trial (figure 2G, H). HRs were also evaluated using sPD-L1 as a continuous variable, and no significant associations were seen (online supplemental file 2G, H).

### Association between baseline sPD-L1 level and gene expression in RCC and melanoma

Gene expression data from baseline biopsies was available for 59 patients in CheckMate 009,^16^ all of whom have baseline sPD-L1 data, and for 49 patients in CheckMate 038-P1 of whom 44 have baseline sPD-L1 data. These gene expression datasets allow evaluation of the relationship between sPD-L1 and transcriptional classifiers that reflect molecular characteristics of cancer (online supplemental table S2). We calculated scores for relevant published signature gene sets and estimated the abundance of immune cell populations using CIBERSORT. Since ADAM proteases have been reported to proteolytically cleave PD-L1 from the surface of breast cancer cells,^27^ we examined expression of 60 such metalloproteases. For CheckMate 009, biopsies were also assigned to RCC molecular subtypes, using the ‘ccrcc’ transcriptional groups (6).

**Figure 3:**
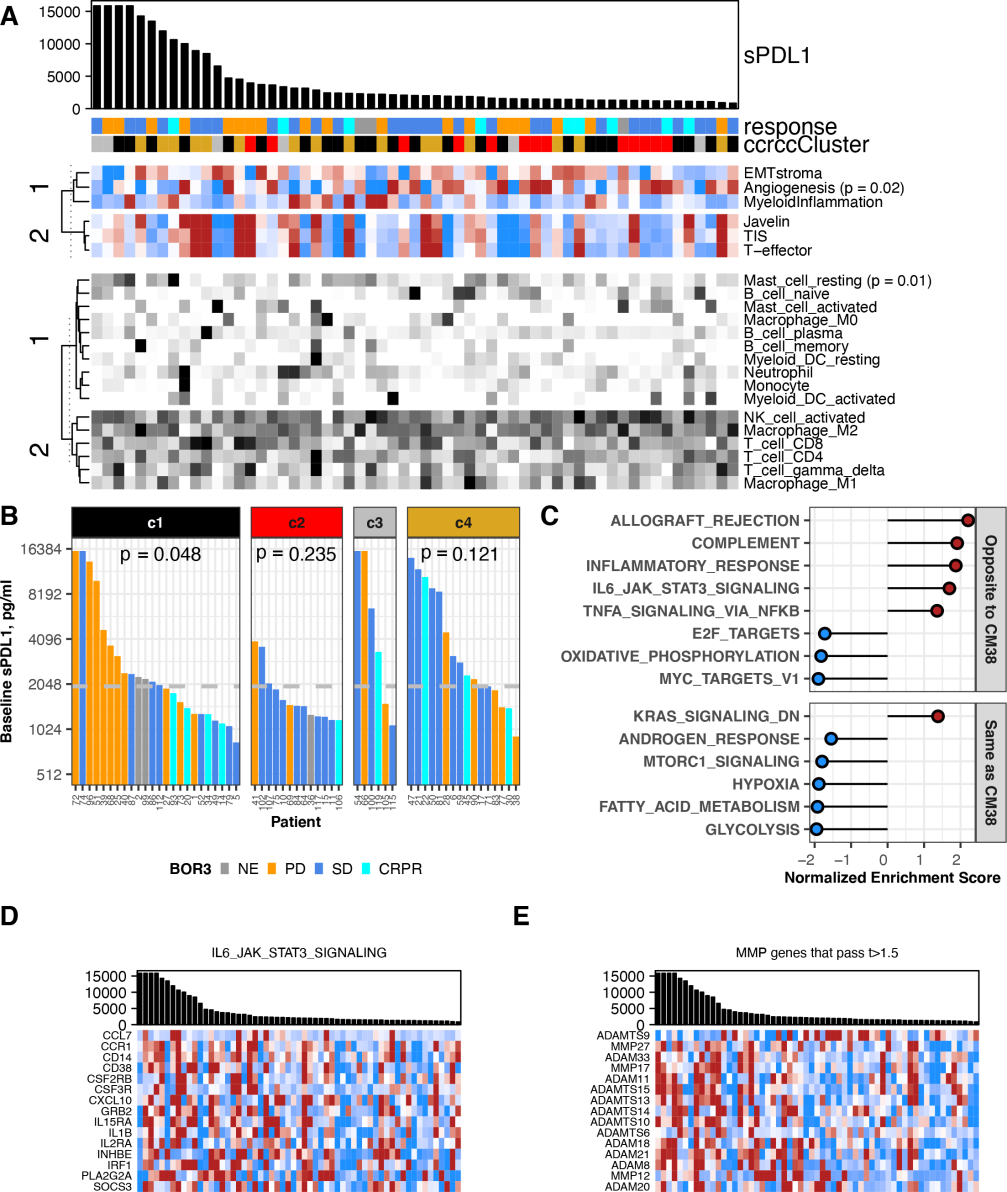
Association between baseline sPD-L1 and gene expression in RCC. All data shown are from 59 patients with a baseline value for sPD-L1 and gene expression data from a baseline biopsy in CheckMate 009. BOR is indicated by gold (PD), blue (SD), aqua (CR or PR) or gray (ND). (A) Biopsy samples are ordered by sPD-L1 level at baseline, provided in the barchart. sPD-L1 values from patients with PD as best response are indicated in gold. Upper heat-map panel shows scores for the gene sets indicated, clustered by their similarity. Scale is −1 to 1 (blue to red). Lower heat-map panel shows CIBERSORT values for the immune cell types indicated, clustered by their similarity. Scale is 0–0.5 (white to black). Sample annotation track shows predicted ccrcc subtype for the biopsy (ccrcc1 as black, ccrcc2 as red, ccrcc3 as gray, and ccrcc4 as gold). (B) Barchart shows sPD-L1 level at baseline. Data are grouped by assigned ccrcc subtype for the biopsy. The median value of sPD-L1 at baseline in all patients (1978 pg/mL) is indicated by a gray horizontal line. P values are from Kruskal-Wallis rank sum test for distribution of BOR in each ccrcc sybtype. (C) Normalized enrichment score from GSEA evaluating Hallmark pathway gene sets in the results for differential gene expression associated with baseline sPD-L1 level. Plot shows 14 pathways that were associated with sPD-L1 level in both CheckMate 009 and 038. Pathways showing the same direction of association are labeled in red. (D) Barchart shows sPD-L1 level at baseline. Heat-map panel shows z-scored expression data for the 15 transcripts from the Hallmark pathway ‘IL6 JAK STAT3 Signaling’ that were associated with baseline sPD-L1 level in both CheckMate 009 and 038. Scale is −1 to 1 (blue to red). (E) Barchart shows sPD-L1 level at baseline. Heat-map panel shows z-scored expression data for the 15 transcripts encoding metalloprotease enzymes that were associated with baseline sPD-L1 level in CheckMate 009 with t-value >1.5. Scale is −1 to 1 (blue to red). BOR, best overall response; CR, complete response; GSEA, Gene Set Enrichment Analysis; PD, progressive disease; PR, partial response; RCC, renal cell carcinoma; SD, stable disease.

In CheckMate 009/RCC, baseline sPD-L1 level had a negative association with the Angiogenesis transcriptional score, and positive association with presence of resting mast cells (p<0.05, figure 3A, online supplemental figure 3A, B and table S3). We also observed significant association between baseline sPD-L1 and our assigned ‘ccrcc’ molecular subtype, which persisted at on-treatment time points (figure 3B, (online supplemental figure 3C, D). Specifically, baseline sPD-L1 values were consistently lower in patients with ccrcc2-subtype RCC (p=0.01 vs ccrcc4, (online supplemental figure S3E). Also, in patients with above median sPD-L1, the patients of ccrcc1 subtype were predominantly PD, whereas the patients of ccrcc4 subtype were predominantly SD and CRPR. This association between sPD-L1 level and BOR is significant in ccrcc1-subtype patients at baseline (p=0.048; figure 3B) and day 63 (p=0.018; online supplemental figure 3D). To investigate the relationship of baseline sPD-L1 level to other transcriptional characteristics in CheckMate 009/RCC, we performed differential gene expression analysis for 18,562 genes followed by GSEA to identify biological processes (figure 3C, online supplemental figure 3F). Among the Hallmark gene sets, several immune-related processes including ‘IL6/Jak/Stat3 Signaling’ were expressed at higher levels in biopsies from patients with high baseline sPD-L1 (figure 3C, D). Conversely, gene sets for metabolic and proliferative processes (‘Oxidative Phosphorylation’, ‘MYC targets V1’) were expressed at lower mean levels in patients with high baseline sPD-L1 (figure 3C). For the majority of the 60 MMP/ADAM genes, we observed that transcripts were higher in patients where sPD-L1 levels were higher, including 14 of the 15 most significantly associated transcripts (t-values >1.5; figure 3E, online supplemental table S7).

**Figure 4:**
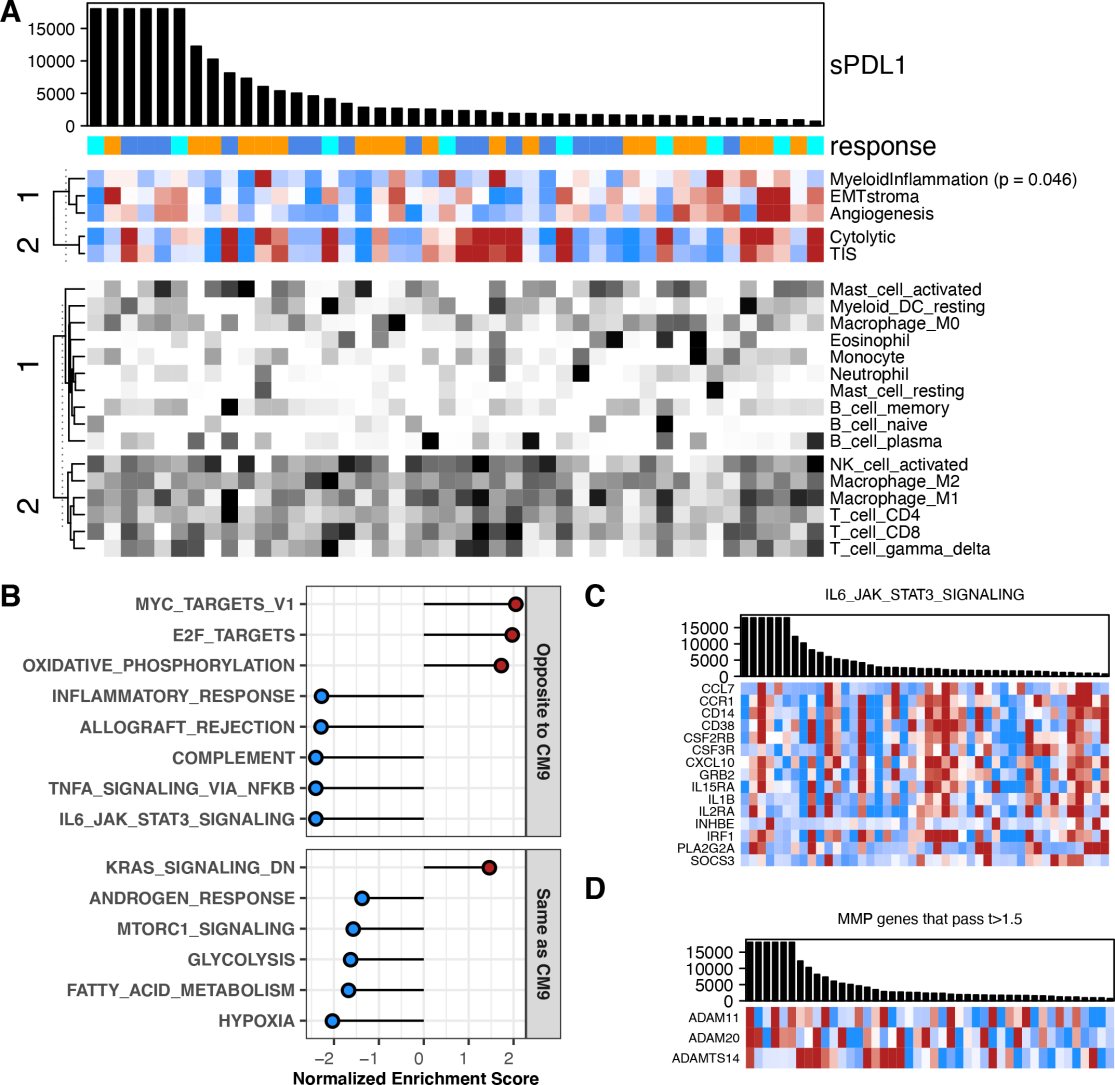
Association between baseline sPD-L1 and gene expression in melanoma. All data shown are from 44 patients with a baseline value for sPD-L1 and gene expression data from a baseline biopsy in CheckMate 038-P1. BOR is indicated by gold (PD), blue (SD) or aqua (CR or PR). (A) Biopsy samples are ordered by sPD-L1 level at baseline, provided in the barchart. sPD-L1 values from patients with PD as best response are indicated in gold. Upper heat-map panel shows scores for the gene sets indicated, clustered by their similarity. Scale is −1 to 1 (blue to red). Lower heat-map panel shows CIBERSORT values for the immune cell types indicated, clustered by their similarity. Scale is 0–0.5 (white to black). (B) Normalized enrichment score from GSEA evaluating Hallmark pathway gene sets in the results for differential gene expression associated with baseline sPD-L1 level. Plot shows 14 pathways that were associated with sPD-L1 level in both CheckMate 009 and 038. Pathways showing the same direction of association are labeled in red. (C) Barchart shows sPD-L1 level at baseline. Heat-map panel shows z-scored expression data for the 15 transcripts from the Hallmark pathway ‘IL6 JAK STAT3 Signaling’ that were associated with baseline sPD-L1 level in both CheckMate 009 and 038. Scale is −1 to 1 (blue to red). (D) Barchart shows sPD-L1 level at baseline. Heat-map panel shows z-scored expression data for the three transcripts encoding metalloprotease enzymes that were associated with baseline sPD-L1 level in CheckMate 038-P1 with t-value >1.5. Scale is −1 to 1 (blue to red). BOR, best overall response; CR, complete response; GSEA, Gene Set Enrichment Analysis; PD, progressive disease; PR, partial response; SD, stable disease.

In CheckMate 038-P1/melanoma, higher baseline sPD-L1 level had negative association with the ‘Myeloid Inflammation’ score (p=0.046, figure 4A, online supplemental figure S4A). We saw no association with immune cell presence, although lower sPD-L1 had a non-significant association with higher levels of neutrophils (p=0.07, figure 4A, online supplemental figure S4B, table S4). To investigate the relationship of baseline sPD-L1 level to other transcriptional characteristics of CheckMate 038-P1/melanoma, we performed differential gene expression analysis for 18 562 genes followed by GSEA to identify biological processes (figure 4B, online supplemental figure S4C). Among the Hallmark gene sets, several metabolic and proliferative process sets (‘oxidative phosphorylation’, ‘MYC targets V1’) were expressed at higher levels in biopsies from patients with high baseline sPD-L1 (figure 4C). Conversely, gene sets for several immune-related processes including ‘IL6/Jak/Stat3 Signaling’ were expressed at lower mean levels in patients with high baseline sPD-L1 (figure 4C). There was no predominant direction of association across the 60 MMP/ADAM genes, however, the three most significantly associated MMP/ADAM transcripts were higher in patients where sPD-L1 levels were higher (t-values >1.5; figure 4D, online supplemental table S7).

Six Hallmark gene sets were enriched with the same direction in GSEA results from both RCC and melanoma: high baseline sPD-L1 is associated with lower expression of genes from ‘Hypoxia’, ‘Fatty Acid Metabolism’, ‘Glycolysis’, ‘MTORC1 signaling’ and ‘Androgen Response’, and with higher expression of genes from ‘KRAS signaling_Down’. A further eight Hallmark gene sets were enriched but with the opposite direction in melanoma and RCC (figures 3C and 4B, online supplemental table S5). These eight gene sets include several immune-related processes such as ‘IL6/Jak/Stat3 Signaling’ that are associated with high baseline sPD-L1 in CheckMate 009 but low baseline sPD-L1 in CheckMate 038-P1 (figures 3D and 4C). Core enrichment genes shared between the RCC and melanoma datasets are presented in online supplemental table S6. With respect to the relationship between the level of baseline sPD-L1 and transcripts encoding 60 metalloproteases, ADAM11, ADAM20 and ADAMTS14 were identified as associated with higher baseline sPD-L1 levels in both CheckMate 009/RCC and CheckMate 038-P1/melanoma (t-values >1.5, figures 3E and 4D, online supplemental table S7).

### Association between change in sPD-L1 on therapy and clinical outcome

In CheckMate 009/RCC and CheckMate 038-P1/melanoma, serum samples were evaluated at two on-treatment timepoints, allowing investigation of the relationship between change in sPD-L1 and outcome. These analyses of paired samples excluded any cases where sPD-L1 values exceeded the ULOQ. It should be noted that patients with PD are under-represented at the later on-treatment timepoint, reducing our ability to detect association (table 1).

**Figure 5:**
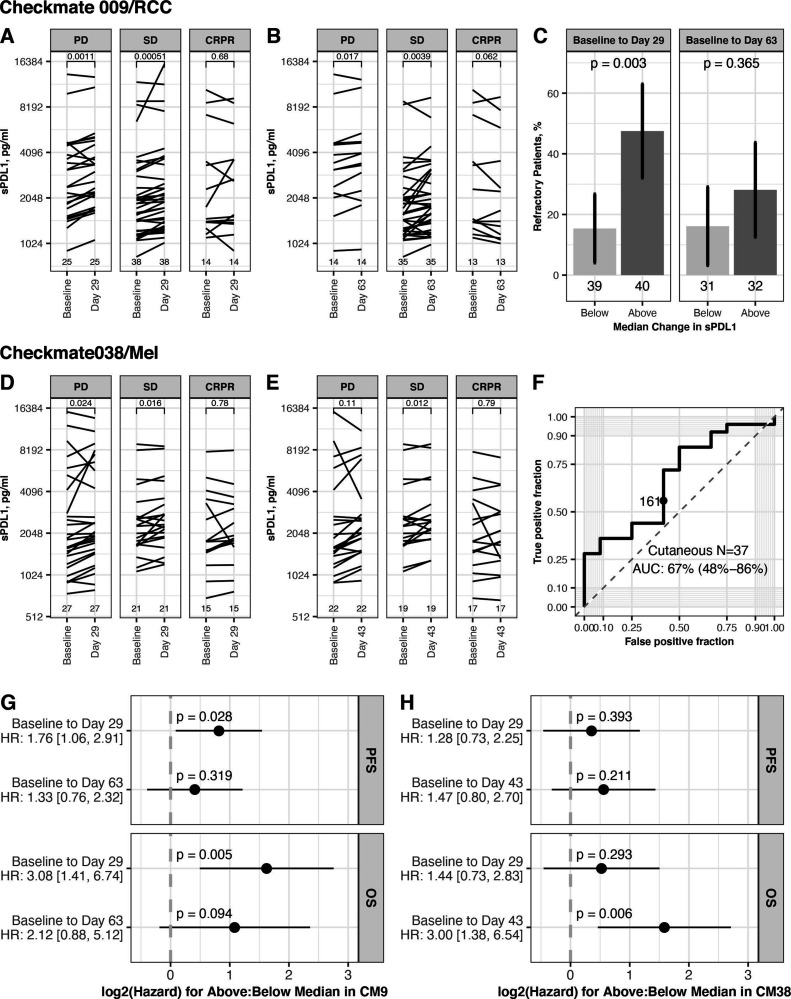
Association between change in sPD-L1 on therapy and outcome. Data shown are for patients from CheckMate 009 (upper panels) and CheckMate038 (middle panels). Partition at the median change uses sPD-L1 values given in table 1. (A) Level of sPD-L1 at baseline and day 29 in CheckMate 009 patients with both values (n=79), grouped by BOR. Lines connect values for each patient. P values from paired t-test (two sided). (B) Level of sPD-L1 at baseline and day 63 in CheckMate 009 patients with both values (n=63), grouped by BOR. Lines connect values for each patient. P values from paired t-test (two sided). (C) Rate of refractory patients (best response of PD in CheckMate 009) in groups based on partition at the median change of sPD-L1. Data presented using partition by median change in sPD-L1 at Day 29 (n=79) and median change at day 63 (n=63). P values from Fisher’s exact test. (D) Level of sPD-L1 at baseline and day 29 in CheckMate 038-P1 patients with both values (n=79), grouped by BOR. Lines connect values for each patient. P values from paired t-test (two sided). (E) Level of sPD-L1 at baseline and day 63 in CheckMate 038-P1 patients with both values (n=63C), grouped by BOR. Lines connect values for each patient. P values from paired t-test (two-sided). (F) ROC curve summarizing predictive accuracy for the change in sPD-L1 at day 43 in CheckMate 038 patients with cutaneous melanoma (n=37, AUC=67%, 95% CI 48% to 86%). The observation nearest the median value of change in sPD-L1 at Day 43 in all patients (171 pg/mL) is indicated. (G) Cox proportional hazard analysis of survival in CheckMate 009, comparing patients based on partition at the median change of sPD-L1, for each timepoint indicated. Panel displays p value and zero-centered HR. HR and 95% CIs are indicated to left. (H) Cox proportional hazard analysis of survival in CheckMate 038-P1, comparing patients based on partition at the median change of sPD-L1, for each timepoint indicated. Panel displays p value and zero-centered HR. HR and 95% CIs are indicated to left. AUC, area under curve; BOR, best overall response; CR, complete response; OS, overall survival; PFS, progression-free survival; PD, progressive disease; PR, partial response; SD, stable disease.

In CheckMate 009/RCC, most patients showed an increase in sPD-L1 on therapy at day 29 and at day 63 (median change 180 pg/mL and 114 pg/mL, respectively; table 1). We saw associations between the increase in sPD-L1 and BOR. The increase of sPD-L1 was greatest in PD, both at day 29 (median change +259 pg/mL) and at day 63 (median change +224 pg/mL) (table 1). We saw a significant increase in sPD-L1 in patients with PD and SD but not in patients with CRPR, at day 29 (p=0.001, 0.001 and 0.68, respectively, figure 5A) and at day 63 (p=0.017, 0.004 and 0.062, respectively, figure 5B). A greater change in sPD-L1, based on partition at the median change, was associated with an increased percentage of refractory patients (best response of PD). This association is significant at day 29, where 47.5% of patients with above median increase are refractory (p=0.003, figure 5C). The predictive accuracy of change in sPD-L1 for Response (as defined in reference^19^) was also evaluated by ROC analysis. Change at Day 63 had predictive accuracy, with an AUC of 67% (95% CI 50% to 85%) (online supplemental figure S5A).

In CheckMate 038-P1/melanoma, most patients showed an increase in sPD-L1 on therapy at day 29 and at day 43 (median change +175 pg/mL and 171 pg/mL, respectively). There was an association between the increase in sPD-L1 and BOR. The increase of sPD-L1 was least in CRPR, both at day 29 (median change +79 pg/mL) and at day 43 (median change +70 pg/mL) (table 1). At day 29, patients with CR+PR did not have a significant increase in sPD-L1, while patients with PD and SD had a significant increase (p=0.024, 0.016 and 0.78 for PD, SD, CR+PR, respectively, figure 5D). At day 43, patients with SD had a significant increase (p=0.11, 0.012 and 0.79 for PD, SD, CR+PR, respectively, figure 5E). A greater change in sPD-L1 at day 43, based on partition at the median change, had non-significant association with an increased percentage of refractory patients (p=0.18, online supplemental figure S5B). The predictive accuracy of change in sPD-L1 was also evaluated by ROC analysis. Change at day 43 had some predictive accuracy for Objective Response, with AUC of 67% in patients with cutaneous melanoma and 64% in the entire cohort (figure 5F, online supplemental figure S5C).

We also evaluated the association between change in sPD-L1 and survival, and observed that increase of sPD-L1 on therapy is related to worse survival outcome both in CheckMate 009/RCC and in CheckMate 038-P1/melanoma (figure 5, online supplemental figure S5C). This relationship achieved statistical significance in several instances, although these trials were not designed to evaluate late clinical outcomes. In CheckMate 009/RCC, patients with above-median change at Day 29 (ie, an increase >180 pg/mL) had a significant increase in hazard both for PFS (HR: 1.76 [1.06, 2.91]) and for OS (HR 3.08, 95% CI 1.41 to 6.74; figure 5G). In CheckMate 038-P1/melanoma, patients with above median change at Day 43 (ie, an increase >171 pg/mL) had a significant increase in hazard for OS (HR 3.00, 95% CI 1.38 to 6.54; figure 5H). Analyses using change in sPD-L1 as a continuous variable also found significant association (online supplemental figure S5D, E).

### Association between change in sPD-L1 and gene expression

Having observed an association between increase of sPD-L1 on therapy and poor clinical outcomes, in both CheckMate 009/RCC and CheckMate 038-P1/melanoma, we wished to further characterize the biological basis of the increase. We first compared the change in sPD-L1 at Day 29 to the change in Cibersort immune cell populations in pretreatment vs day 29 biopsies (online supplemental tables S8,9). We did not see any significant associations, although in CheckMate 009/RCC, patients with greater sPD-L1 increase generally had decreases in neutrophil counts (p=0.07, online supplemental figure 6A).

**Figure 6:**
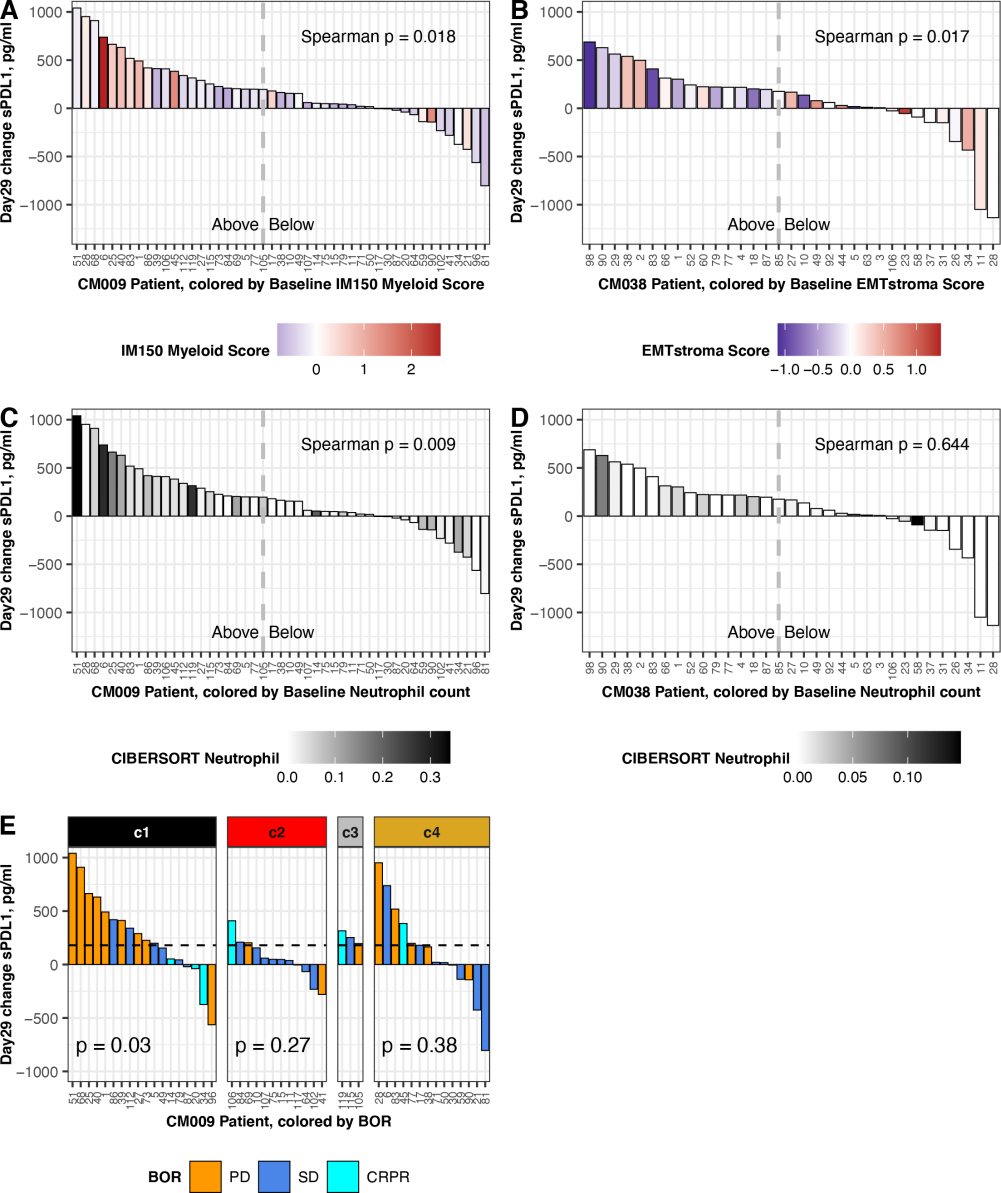
Association between change in sPD-L1 and baseline gene expression. Data shown are from patients with baseline biopsy gene expression and a day 29 change result for sPD-L1. Left panels show data from CheckMate 009 (n=47; three outliers were omitted: 9159, 1887,–1943 pg/mL). Right panels show data from CheckMate 038-P1 (n=33; two outliers were omitted: 5280, 1769 pg/mL). Dashed lines indicate the median value of day 29 sPD-L1 change in the complete patient cohort: 180 pg/mL in CheckMate 009 and 175 pg/mL in CheckMate 038-P1 (table 1). (A) Change in sPD-L1 at day 29 in CheckMate 009, colored by Myeloid Inflammation Score in biopsy from respective patient. Scale is −1 to 2.5 (blue to red). (B) Change in sPD-L1 at day 29 in CheckMate 038-P1, colored by EMT/Stroma Score in biopsy from respective patient. Scale is −1 to 1.5 (blue to red). (C) Change in sPD-L1 at day 29 in CheckMate 009, colored by CIBERSORT value for Neutrophils in biopsy from respective patient. Scale is 0–0.35 (white to black). (D) Change in sPD-L1 at Day 29 in CheckMate 038-P1, colored by CIBERSORT value for Neutrophils in biopsy from respective patient. Scale is 0–0.15 (white to black). (E) Change in sPD-L1 at Day 29 in CheckMate 009. Data are partitioned by the assigned ccrcc subtype from the tumor biopsy. BOR is indicated by gold (PD), blue (SD) or aqua (CR or PR). P values are from Kruskal-Wallis rank sum test for distribution of BOR in each ccrcc sybtype. BOR, best overall response; CR, complete response; PD, progressive disease; PR, partial response; SD, stable disease.

We then analyzed correlation between our scores for published transcriptional gene sets, derived in pretreatment biopsies, and the change in sPD-L1 at day 29 of therapy (online supplemental tables S10,11). We found two significant associations: in CheckMate 009/RCC, patients with higher Myeloid Inflammation score at baseline experienced significantly greater increase in sPD-L1 (p=0.018, figure 6A), and in CheckMate 038-P1/melanoma, patients with lower EMT/stroma score at baseline experienced significantly greater increase in sPD-L1 (p=0.017, figure 6B).

We also analyzed correlation between the Cibersort immune cell estimates in pretreatment biopsies and the change in sPD-L1 at day 29 of therapy (online supplemental tables S10,11). In CheckMate 009/RCC we saw significant positive association between baseline neutrophil count and sPD-L1 increase (p=0.009, figure 6C) and a negative association with activated NK cell count (p=0.018, online supplemental figure S6B). In CheckMate 038/melanoma, the Cibersort baseline counts for neutrophils and activated NK cells were lower than in CheckMate 009/RCC (maxima of 0.15 and 0.08, vs 0.34 and 0.19, respectively). Although a positive association between sPD-L1 increase and baseline neutrophil count and a negative association with baseline activated NK cell count was also seen in CheckMate 038/melanoma, the values did not meet statistical significance (p=0.6, figure 6D (online supplemental figure S6D).

Finally, we observed that in CheckMate 009/RCC patients with ccrcc1 subtype tumors had the highest median change in sPD-L1 at day 29 (online supplemental figure S6D). We then evaluated whether the association between change in sPD-L1 and BOR (figure 5A) was affected by our assigned ccrcc molecular subtype for the patient. The change in sPD-L1 at day 29 was associated with BOR in the ccrcc1 subtype (p=0.03; figure 6E). When patients were partitioned at the median change in sPD-L1 at Day 29 (ie,+180 pg/mL; table 1), in the ccrcc1 subtype a greater change in sPD-L1 was associated with an increased percentage of refractory disease, whereas in the ccrcc 4 subtype the association was not significant (p=0.05 vs 0.266; online supplemental figure S6E).

## Discussion

We investigated possible associations to sPD-L1 levels in two prospective trials of nivolumab treatment, and found an association with some clinical characteristics, as well as association with PD and worse survival outcomes in both trials. This study is the first to report an association between serum sPD-L1 levels and clinical outcomes on nivolumab before and during treatment of metastatic ccRCC and metastatic melanoma. Furthermore, the association of PD with change in sPDL1 on treatment is the first biomarker to agree between melanoma and RCC.

Our findings are consistent with sPD-L1 being a marker of aggressive disease in both RCC and melanoma,^14^ since high sPD-L1 is associated with having progressed from prior VEGFi therapy in patients with RCC and with high LDH (a marker of poor prognosis) in patients with melanoma. While single-agent therapeutic trial analysis, such as for CheckMate 009 and 038-P1, cannot be used to distinguish a predictive marker from a prognostic biomarker, our findings clearly show that sPD-L1 is likely a complex marker and not simply a surrogate for PD-L1 expressing tumors. Our findings suggest that nivolumab-refractory disease produces sPD-L1, but there may be a distinct secondary pathway by which some patients with CR or PR on nivolumab produce sPD-L1. The comparison of the analysis of expression array data between the two tumor types found an association of high sPD-L1 with many immunological pathways in kidney cancer, not seen in melanoma. There was no selection bias on the conclusions from our analysis of baseline blood samples, since 100% of baseline blood samples were tested in CheckMate 009% and 90% (78/87) in CheckMate 038-Part1, as illustrated in the schematic in figure 1. While blood samples were collected after progression, the decrease of samples was largely due to disease progression (online supplemental table 1). High baseline sPD-L1 trended with PD on nivolumab in patients with RCC, but in melanoma, high baseline levels of sPD-L1 were not associated with clinical outcomes. In CheckMate 009/RCC, sPD-L1 was higher on average at all timepoints in patients with PD relative to SD (figure 2A–C). sPDL1 may be a marker for tumor burden of PD-L1-positive disease, and/or sPD-L1 may be a surrogate for other mechanisms of immunotherapy-resistant disease, such as a protumorigenic microenvironment. Not only have myeloid cells been shown to produce sPD-L1,^13^ but our RNA analysis identified three matrix metalloprotease transcripts that are associated with high sPD-L1 in both RCC and melanoma (ADAM11, ADAM20, and ADAMTS14; figures 3E and 4D). This finding in melanoma is consistent with the prior report that sPD-L1 was not associated with response to PD-1 blockade, though in that report exosomal PD-L1 increased on treatment in clinical responders.^28^

Our analysis of gene expression in pretreatment biopsies from RCC found a clear association between low baseline sPD-L1 and higher expression of an ‘Angiogenesis’ signature that predicts a positive response to sunitinib in the ImMotion 150 trial.^4^ The ‘Angiogenesis’ signature also predicts refractory disease in CheckMate 009/RCC.^19^ Thus sPD-L1 in the peripheral blood may be a more accessible, less expensive surrogate for ‘Angiogenesis’ signature of RCC tumors. Interestingly, high baseline sPD-L1 is associated with higher expression of genes from ‘KRAS signaling_Down’ and lower expression of genes from ‘Hypoxia’, ‘Fatty Acid Metabolism’, ‘Glycolysis’, ‘MTORC1 signaling’ and ‘Androgen Response’ processes in both RCC and melanoma, suggesting that high sPD-L1 is distinct from a number of potentially targetable pathways. In this relatively small hypothesis-generating study, high sPD-L1 levels may not be a strong predictor of response to nivolumab in RCC as a solitary marker, but pretreatment sPD-L1 in conjunction with molecular ccrcc clustering/subtyping may prove to be a clinically useful biomarker for nivolumab-refractory disease in RCC. This is further supported by our differentially expressed genes (DEG) analysis, which revealed fourteen genes (CD36, NDRG1, SDC4, PDK1, VEGFA, CXCR4, ERRFI1, CCND1, MAP3K1, TNFAIP3, INPP4B, SOX9, RIT1, and ALOX12B) that were considered potentially actionable by the Foundation One or MSK-IMPACT testing databases (online supplemental table S6). With the exception of ALOX12B, higher baseline expression of these actionable genes correlated with low sPD-L1. Specifically, VEGFA and NDRG1 are targetable with VEGFR-TKI, and are thus relevant for patients with RCC. In the rapidly evolving landscape of RCC therapy, several PD-1 based combinations have been approved in the last 4 years for patients with advanced kidney cancer, with either CTLA4 blockade or VEGF tyrosine kinase inhibition.^29–32^ Given the poor outcomes in patients with high baseline sPD-L1 and that low sPD-L1 is associated with the ‘Angiogenic’ signature associated with response to VEGF tyrosine kinase inhibition, the level of baseline sPD-L1 may be a useful means of deciding between first-line PD-1 combination therapies, that is, PD-1 with CTLA4 blockade in patients with high sPD-L1 and PD-1 blockade with VEGF tyrosine kinase inhibition in patients with low sPD-L1. Expression of six Hallmark gene sets was associated with sPDL1 level in the same direction in both RCC and melanoma, indicating a shared underlying tumor biology. Eight Hallmark gene sets were enriched but with the opposite direction for association with sPD-L1 in the two tumor types (figures 3C and 4B). The latter phenomena is particularly interesting since five of the Hallmark sets that show opposite association with sPD-L1 reflect inflammatory processes. Since both melanoma and RCC are considered inflamed tumors, this data suggests that high sPD-L1 may be associated with different categories of inflammation (eg, IL6, TNFA, or complement-dependent) depending on the tumor type.

Interestingly, on nivolumab therapy, an increase in sPD-L1 is associated with progression in both RCC and melanoma, suggesting a potential immunologic mechanism of early resistance to PD-1 blockade in both tumor types. sPD-L1 is a noninvasive means of monitoring the early trajectory of the disease on treatment. While the association between the sPD-L1 increase at day 29 and lack of response (PD or SD) was significant in both trials, day 29 is not an optimized time point. Analysis of a full time series could refine the performance of sPDL1 as an indicator of PD. In the CheckMate 009/RCC study, prior investigations focused on exploring predictors of response to nivolumab failed to find a clinically useful predictive biomarker, which may have been due in part to the study having a low response rate relative to other nivolumab clinical trials in kidney cancer.^16 19^ However, in CheckMate 009/RCC, the high rate of early PD provides greater potential to discover clinically useful markers of rapid progression in our exploratory analysis. Additional validation will be required to confirm whether high sPD-L1 will define a subset of patients with ccrcc1 subtype of RCC that will rapidly progress on PD-1 blockade. Of note, in BIONIKK, the patients with ccrcc1 tumors had the greatest benefit from the addition of ipilimumab.^33^ As combination therapies rapidly become the standard of care in kidney cancer, sPD-L1 levels may refine our understanding of who may benefit from which combination therapies in a multiomic manner. Larger cohorts of RCC will need to be studied to determine whether baseline or the early increase in sPD-L1 on therapy may augment the molecular ccrcc clustering to create a clinically useful early marker for patients at greatest risk for nivolumab-refractory disease.

## Data Availability

PD-L1 data and Affymetrix data are available in public, open access repositories.
