# Supplementary material for: Soluble PD-L1 as an early marker of progressive disease on nivolumab
Source: J Immunother Cancer. 2022 Feb 7;10(2):e003527. doi: 10.1136/jitc-2021-003527 (PMC8823247; doi:10.1136/jitc-2021-003527)
Supplement: Supplementary data [file jitc-2021-003527supp002.pdf]

Online Supplemental Figures

Figure S1. Association between baseline sPD-L1 and clinical characteristics

Serum level of sPD-L1 Samples provided at Baseline in CheckMate 009 (n = 91, upper panels) and CheckMate 038-P1 (n = 78, lower panels) are indicated by circles. For samples where sPDL1 level exceeded the ULOQ, the imputed values are indicated by square symbols. All *p*-values derived from Wilcoxon Rank Sum test.

a. CheckMate 009 data are grouped by patients who are less than 65 years old (<65y, n = 61) or patients ≥ 65 years old (>=65y, n = 30).
b. CheckMate 009 data are grouped by patients who are male (n = 61) or female (n = 30).
c. CheckMate 009 data are grouped by patients who have a mutation ("Nonsense_Mutation", "Missense_Mutation", "Splice_Site", "Frame_Shift_Del", "Frame_Shift_Ins") in PBRM1 (MUT = 18), no mutation (WT, n = 33), or no genomic status data (ND, n = 40).
d. CheckMate 009 data are grouped by patients who have a mutation ("Nonsense_Mutation", "Missense_Mutation", "Splice_Site", "Frame_Shift_Del", "Frame_Shift_Ins") in VHL1 (MUT = 28), no mutation (WT, n = 23), or no genomic status data (ND, n = 40).
e. CheckMate 038 data are grouped by patients who are less than 65 years old (<65y, n = 61) or patients ≥ 65 years old (>=65y, n = 17).
f. CheckMate 038 data are grouped by patients who are male (n = 44) or female (n = 34).
g. CheckMate 038 data are grouped by diagnostic subtype: Cutaneous (n = 50), Mucinous (n = 7), Uveal (n = 6), Acral (n = 2) or Other (n = 13).
h. CheckMate 038 data are grouped by patients whose serum LDH at baseline was below the upper limit of normal (<ULN, n = 49) or above >ULN, n = 28). One patient had no LDH category reported.

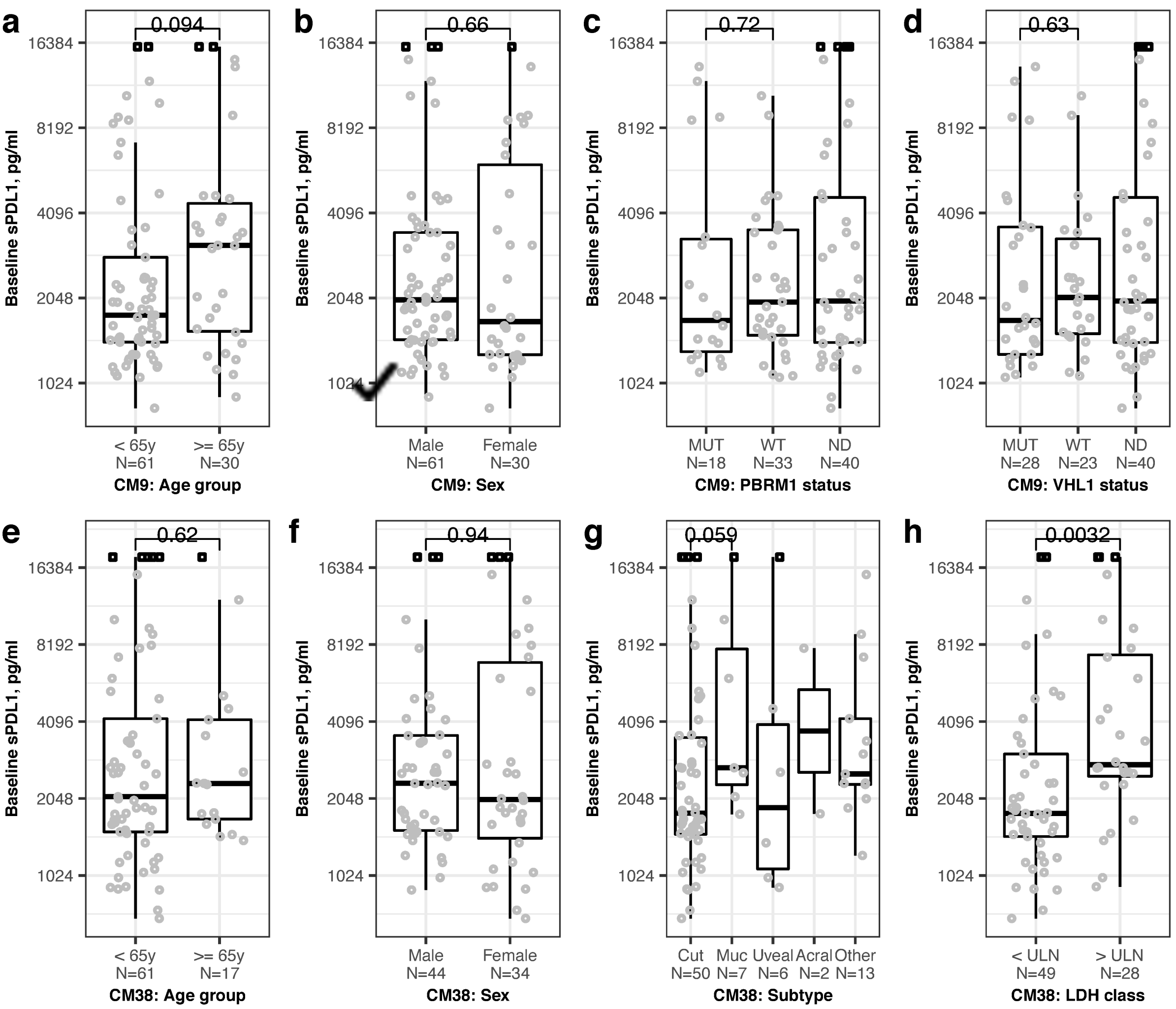
a
0.094
Baseline sPDL1, pg/ml
16384
8192
4096
2048
1024
< 65y N=61
>= 65y N=30
CM9: Age group
b
0.66
Male N=61
Female N=30
CM9: Sex
c
0.72
MUT N=18
WT N=33
ND N=40
CM9: PBRM1 status
d
0.63
MUT N=28
WT N=23
ND N=40
CM9: VHL1 status
e
0.62
< 65y N=61
>= 65y N=17
CM38: Age group
f
0.94
Male N=44
Female N=34
CM38: Sex
g
0.059
Cut N=50
Muc N=7
Uveal N=6
Acral N=2
Other N=13
CM38: Subtype
h
0.0032
< ULN N=49
> ULN N=28
CM38: LDH class

Figure S2. Association between sPD-L1 and Response

Serum level of sPD-L1 in patients from CheckMate 009 (upper panels) and CheckMate038-P1 (middle panels). Data in CheckMate 009 are grouped by Response status which was based on a <20% tumor shrinkage criterion. Data in CheckMate 038-P1 are grouped by Objective Response status (Responder = CR or PR, NonResponder = all other outcomes). BOR is indicated by gold squares (PD), blue circles (SD), aqua triangles (CR or PR) or grey circles (ND). All *p*-values are derived from Wilcoxon Rank Sum test.

a. Samples provided at Baseline in CheckMate 009. Data are grouped by Response status (NonResponder, n = 65, Responder, n = 20).
b. Samples provided at Day 29 in CheckMate 009. Data are grouped by Response status (NonResponder, n = 62, Responder, n = 20).
c. Samples provided at Day 63 in CheckMate 009. Data are grouped by Response status (NonResponder, n = 49, Responder, n = 19).
d. Samples provided at Baseline in CheckMate 038. Data are grouped by Response status (NonResponder, n = 58, Responder, n = 20).
e. Samples provided at Day 29 in CheckMate 038. Data are grouped by Response status (NonResponder, n = 52, Responder, n = 17).
f. Samples provided at Day 43 in CheckMate 038. Data are grouped by Response status (NonResponder, n = 45, Responder, n = 19).
g. Cox proportional hazard analysis of survival in CheckMate 009, comparing risk for patients at the 75th percentile of sPDL1 values to patients at the 25th percentile of values, for each timepoint indicated. Essentially this is a test of relative hazard for patients whose sPDL1 values differ by the inter-quartile range. Panel displays *p* value and zero-centered Hazard Ratio. Hazard ratio and 95% confidence intervals are indicated to left.
h. Cox proportional hazard analysis of survival in CheckMate 038, comparing risk for patients at the 75th percentile of sPDL1 values to patients at the 25th percentile of values, for each timepoint indicated. Essentially this is a test of relative hazard for patients whose sPDL1 values differ by the inter-quartile range. Panel displays *p* value and zero-centered Hazard Ratio. Hazard ratio and 95% confidence intervals are indicated to left.

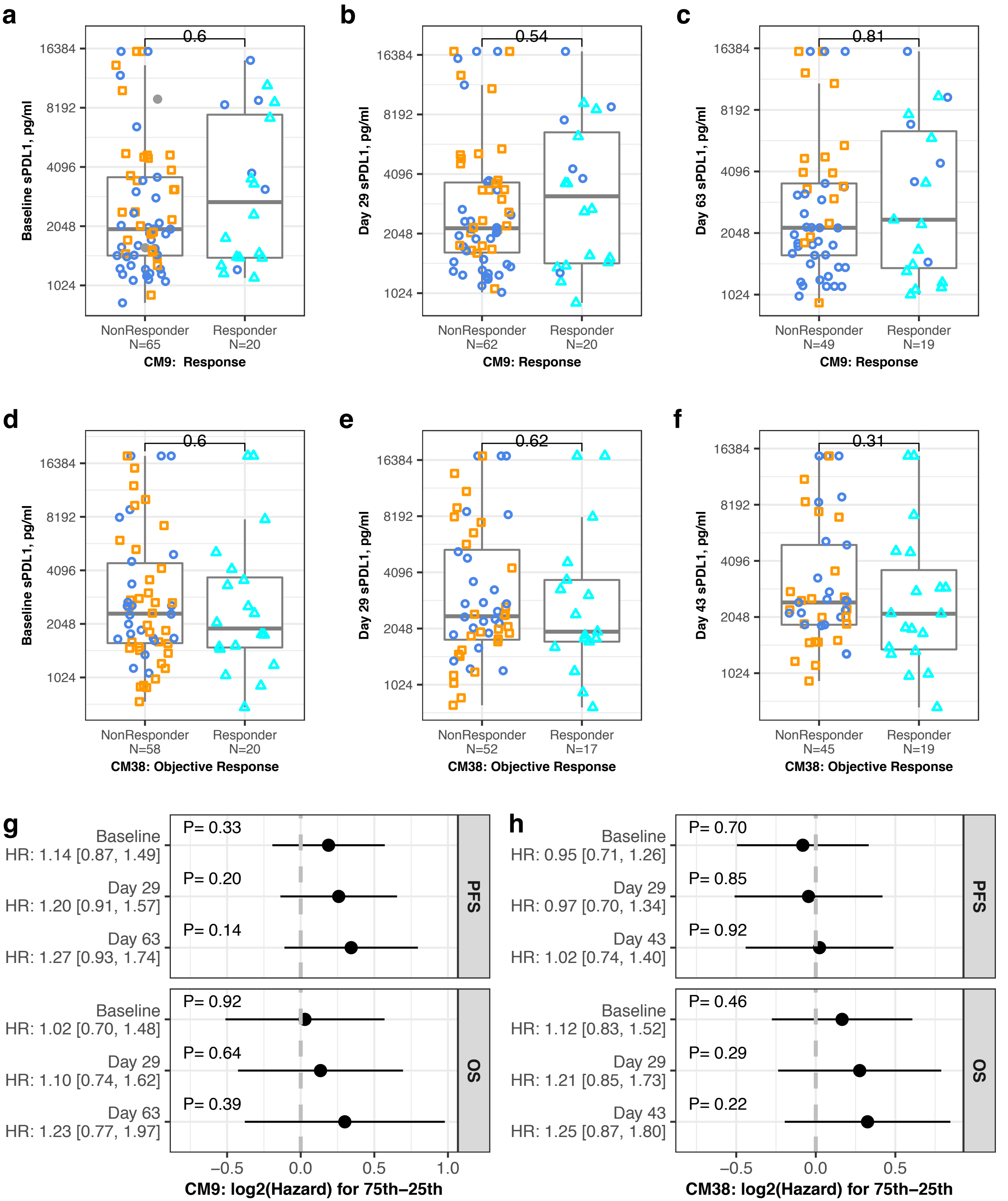
a
b
c
d
e
f
g
h
0.6
0.54
0.81
0.62
0.31
16384
8192
4096
2048
1024
Baseline sPDL1, pg/ml
Day 29 sPDL1, pg/ml
Day 63 sPDL1, pg/ml
Day 43 sPDL1, pg/ml
NonResponder N=65
Responder N=20
NonResponder N=62
NonResponder N=49
Responder N=19
NonResponder N=58
NonResponder N=52
Responder N=17
NonResponder N=45
CM9: Response
CM38: Objective Response
Baseline HR: 1.14 [0.87, 1.49]
P= 0.33
Day 29 HR: 1.20 [0.91, 1.57]
P= 0.20
Day 63 HR: 1.27 [0.93, 1.74]
P= 0.14
Baseline HR: 1.02 [0.70, 1.48]
P= 0.92
Day 29 HR: 1.10 [0.74, 1.62]
P= 0.64
Day 63 HR: 1.23 [0.77, 1.97]
P= 0.39
PFS
OS
−0.5
0.0
0.5
1.0
CM9: log2(Hazard) for 75th−25th
Baseline HR: 0.95 [0.71, 1.26]
P= 0.70
Day 29 HR: 0.97 [0.70, 1.34]
P= 0.85
Day 43 HR: 1.02 [0.74, 1.40]
P= 0.92
Baseline HR: 1.12 [0.83, 1.52]
P= 0.46
Day 29 HR: 1.21 [0.85, 1.73]
P= 0.29
Day 43 HR: 1.25 [0.87, 1.80]
P= 0.22
CM38: log2(Hazard) for 75th−25th

Figure S3. Association between baseline sPD-L1 and gene expression in CheckMate 009/RCC

All data shown are from 59 patients with gene expression data from a baseline biopsy in CheckMate 009. BOR is indicated by gold (PD), blue (SD), aqua (CR or PR) or grey (ND).

a. Patients are ordered by their sPDL1 level at baseline, shown in the barchart. Vertical line indicates the median baseline sPDL1 level calculated on the entire patient cohort. Bars are colored by the Angiogenesis signature score for a baseline biopsy from the same patient. Scale for score is -2 to 1.5 (blue to red).
b. Patients are ordered by their sPDL1 level at baseline, shown in the barchart. Vertical line indicates the median baseline sPDL1 level calculated on the entire patient cohort. Bars are colored by the level of resting Mast cells, determined by Cibersort, in a baseline biopsy from the same patient. Scale for cell content is 0 to 0.15 (white to black).
c. Barchart shows sPDL1 level at Day 29. Data are grouped by ccrcc subtype, predicted from the baseline biopsy. The median value of sPDL1 at Day 29 in the entire patient cohort (2300 pg/ml) is indicated by a grey horizontal line. *P* values for distribution of BOR categories are from the Kruskal-Wallis rank sum test.
d. Barchart shows sPDL1 level at Day 63. Data are grouped by ccrcc subtype, predicted from the baseline biopsy. The median value of sPDL1 at Day 63 in the entire patient cohort (2179 pg/ml) is indicated by a grey horizontal line. *P* values for distribution of BOR categories are from the Kruskal-Wallis rank sum test.
e. Baseline sPDL1 in patients with gene expression data from a baseline biopsy. Data are grouped by ccrcc subtype, predicted from the baseline biopsy: ccrcc1 (n = 24), ccrcc2 (n = 13), ccrcc3 (n = 6), ccrcc4 (n=16). *P* values are from the Wilcoxon test.
f. Normalized enrichment score from GSEA evaluating Hallmark pathway gene sets in the results for differential gene expression associated with baseline sPDL1 level. Plot shows all pathways that were significantly associated with sPDL1 level in CheckMate 009. Pathways that were NOT also identified in CheckMate 038 are labelled in red.

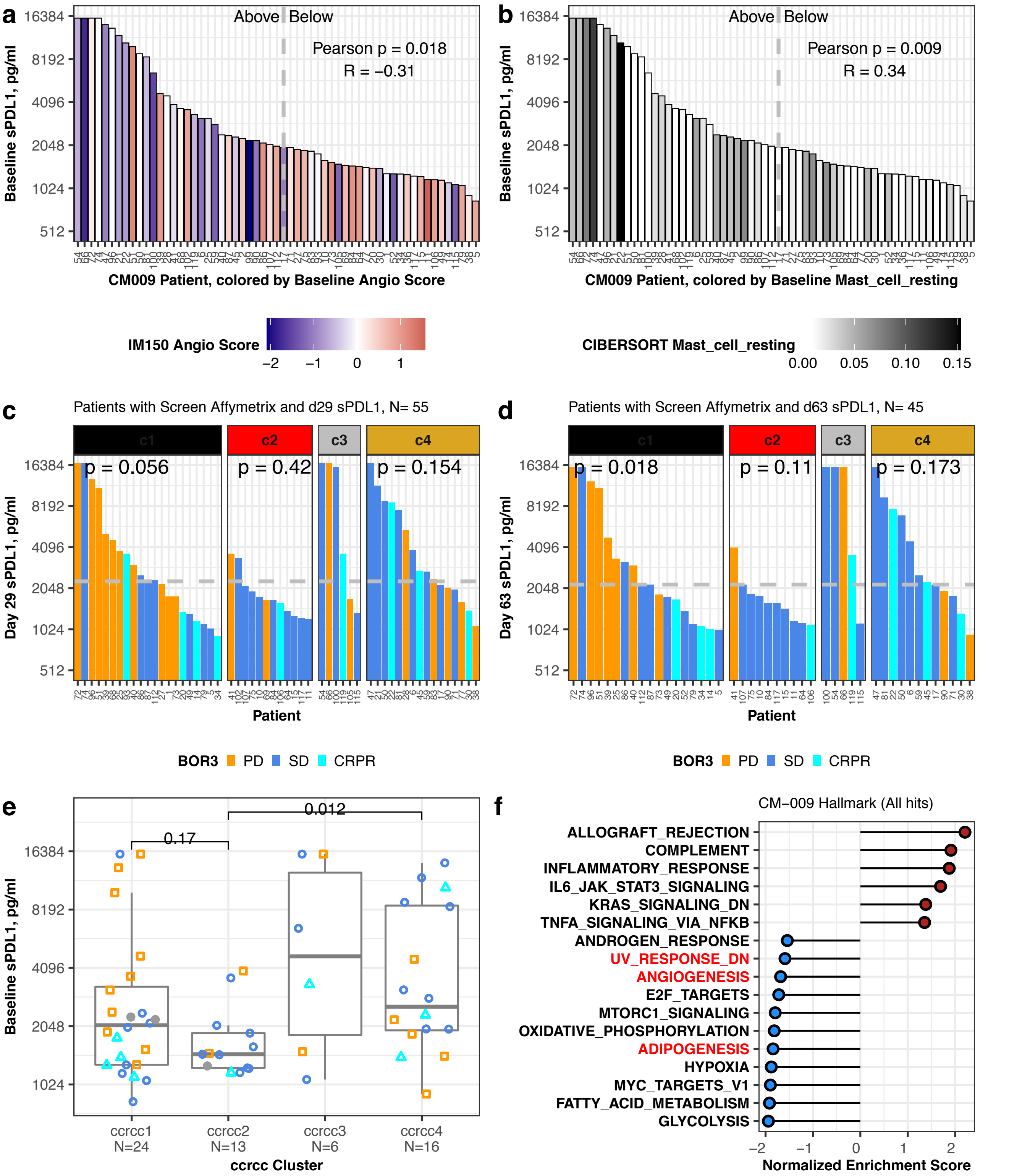
a
Above Below
Pearson p = 0.018
R = −0.31
Baseline sPDL1, pg/ml
16384
8192
4096
2048
1024
512
CM009 Patient, colored by Baseline Angio Score
IM150 Angio Score
−2 −1 0 1
b
Above Below
Pearson p = 0.009
R = 0.34
Baseline sPDL1, pg/ml
CM009 Patient, colored by Baseline Mast_cell_resting
CIBERSORT Mast_cell_resting
0.00 0.05 0.10 0.15
c
Patients with Screen Affymetrix and d29 sPDL1, N= 55
c1 c2 c3 c4
p = 0.056
p = 0.42
p = 0.154
Day 29 sPDL1, pg/ml
Patient
BOR3 PD SD CRPR
d
Patients with Screen Affymetrix and d63 sPDL1, N= 45
c1 c2 c3 c4
p = 0.018
p = 0.11
p = 0.173
Day 63 sPDL1, pg/ml
Patient
BOR3 PD SD CRPR
e
0.17
0.012
Baseline sPDL1, pg/ml
ccrcc1 N=24
ccrcc2 N=13
ccrcc3 N=6
ccrcc4 N=16
ccrcc Cluster
f
CM−009 Hallmark (All hits)
ALLOGRAFT_REJECTION
COMPLEMENT
INFLAMMATORY_RESPONSE
IL6_JAK_STAT3_SIGNALING
KRAS_SIGNALING_DN
TNFA_SIGNALING_VIA_NFKB
ANDROGEN_RESPONSE
UV_RESPONSE_DN
ANGIOGENESIS
E2F_TARGETS
MTORC1_SIGNALING
OXIDATIVE_PHOSPHORYLATION
ADIPOGENESIS
HYPOXIA
MYC_TARGETS_V1
FATTY_ACID_METABOLISM
GLYCOLYSIS
−2 −1 0 1 2
Normalized Enrichment Score

Figure S4. Association between baseline sPD-L1 and gene expression in CheckMate 038-P1/Melanoma

All data shown are from 44 patients with a baseline value for sPDL1 and gene expression data from a baseline biopsy in CheckMate 038-P1. BOR is indicated by gold (PD), blue (SD) or aqua (CR or PR).

a. Patients are ordered by their sPDL1 level at baseline, shown in the barchart. Vertical line indicates the median baseline sPDL1 level calculated on the entire patient cohort. Bars are colored by the Myeloid Inflammation signature score for a baseline biopsy from the same patient. Scale for score is -1 to 3 (blue to red).
b. Patients are ordered by their sPDL1 level at baseline, shown in the barchart. Vertical line indicates the median baseline sPDL1 level calculated on the entire patient cohort. Bars are colored by the level of neutrophils, determined by Cibersort, in a baseline biopsy from the same patient. Scale for cell content is 0 to 0.15 (white to black).
c. Normalized enrichment score from GSEA evaluating Hallmark pathway gene sets in the results for differential gene expression associated with baseline sPDL1 level. Plot shows all pathways that were significantly associated with sPDL1 level in CheckMate 038. Pathways that were NOT also identified in CheckMate 009 are labelled in red.

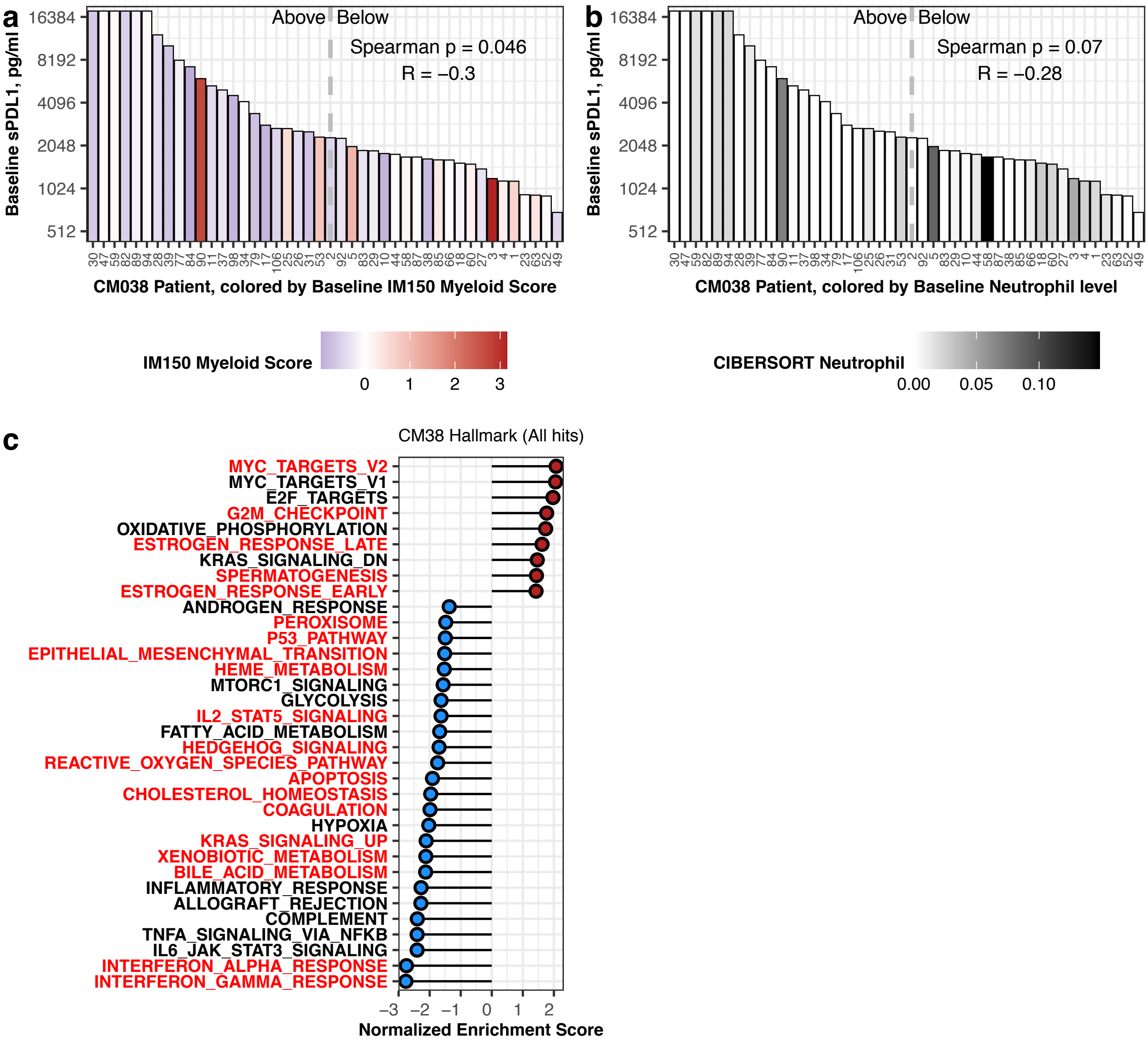
a
Baseline sPDL1, pg/ml
16384
8192
4096
2048
1024
512
Above
Below
Spearman p = 0.046
R = −0.3
CM038 Patient, colored by Baseline IM150 Myeloid Score
b
Baseline sPDL1, pg/ml
Above
Below
Spearman p = 0.07
R = −0.28
CM038 Patient, colored by Baseline Neutrophil level
IM150 Myeloid Score
0
1
2
3
CIBERSORT Neutrophil
0.00
0.05
0.10
c
CM38 Hallmark (All hits)
MYC_TARGETS_V2
MYC_TARGETS_V1
E2F_TARGETS
G2M_CHECKPOINT
OXIDATIVE_PHOSPHORYLATION
ESTROGEN_RESPONSE_LATE
KRAS_SIGNALING_DN
SPERMATOGENESIS
ESTROGEN_RESPONSE_EARLY
ANDROGEN_RESPONSE
PEROXISOME
P53_PATHWAY
EPITHELIAL_MESENCHYMAL_TRANSITION
HEME_METABOLISM
MTORC1_SIGNALING
GLYCOLYSIS
IL2_STAT5_SIGNALING
FATTY_ACID_METABOLISM
HEDGEHOG_SIGNALING
REACTIVE_OXYGEN_SPECIES_PATHWAY
APOPTOSIS
CHOLESTEROL_HOMEOSTASIS
COAGULATION
HYPOXIA
KRAS_SIGNALING_UP
XENOBIOTIC_METABOLISM
BILE_ACID_METABOLISM
INFLAMMATORY_RESPONSE
ALLOGRAFT_REJECTION
COMPLEMENT
TNFA_SIGNALING_VIA_NFKB
IL6_JAK_STAT3_SIGNALING
INTERFERON_ALPHA_RESPONSE
INTERFERON_GAMMA_RESPONSE
−3
−2
−1
0
1
2
Normalized Enrichment Score

Figure S5. Association between change in sPD-L1 upon therapy and Outcome

Data shown are for patients from CheckMate 009 (a,d) and CheckMate 038-P1(b,c,e).

a. ROC curve summarizing predictive accuracy for the change in sPDL1 at day 29 or day 63 in CheckMate 009 patients. The observations closest to the median value of change in sPDL1 in all patients (180 pg/ml at day 29 and 114 pg/ml at day 63) are indicated.
b. Rate of refractory patients (best response of PD in CheckMate 038) in groups based on partition at the median change of sPDL1. Data presented using partition by median change in sPDL1 at Day 29 (n = 63) and median change at Day 63 (n = 58). *p*-values from Fisher exact test.
c. ROC curve summarizing predictive accuracy for the change in sPDL1 at day 29 or day 43 in CheckMate 038 patients. The observations closest to the median value of change in sPDL1 in all patients (175 pg/ml at day 29 and 171 pg/ml at day 43) are indicated.
d. Cox proportional hazard analysis of survival in CheckMate 009, comparing risk for patients at the 75th percentile of sPDL1 values to patients at the 25th percentile of values for sPDL1 change, for each timepoint indicated. Essentially this is a test of relative hazard for patients whose change in sPDL1 values differs by the inter-quartile range. Panel displays *p* value and zero-centered Hazard Ratio. Hazard ratio and 95% confidence intervals are indicated to left.
e. Cox proportional hazard analysis of survival in CheckMate 038, comparing risk for patients at the 75th percentile of sPDL1 values to patients at the 25th percentile of values for sPDL1 change, for each timepoint indicated. Essentially this is a test of relative hazard for patients whose change in sPDL1 values differs by the inter-quartile range. Panel displays *p* value and zero-centered Hazard Ratio. Hazard ratio and 95% confidence intervals are indicated to left.

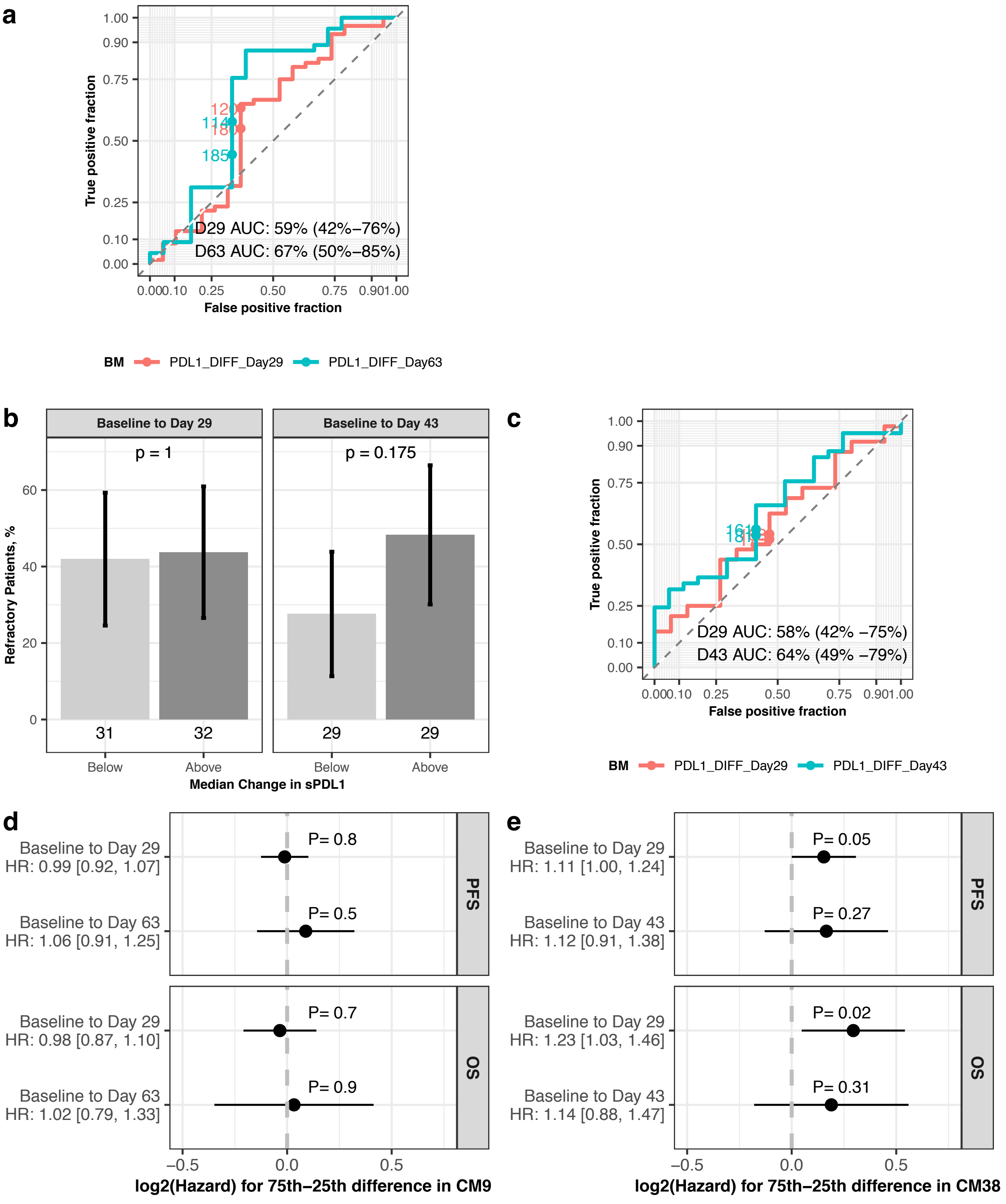
a
True positive fraction
False positive fraction
D29 AUC: 59% (42%–76%)
D63 AUC: 67% (50%–85%)
BM
PDL1_DIFF_Day29
PDL1_DIFF_Day63
b
Baseline to Day 29
Baseline to Day 43
p = 1
p = 0.175
Refractory Patients, %
31
32
29
29
Below
Above
Median Change in sPDL1
c
D29 AUC: 58% (42% –75%)
D43 AUC: 64% (49% –79%)
PDL1_DIFF_Day43
d
Baseline to Day 29
HR: 0.99 [0.92, 1.07]
P= 0.8
Baseline to Day 63
HR: 1.06 [0.91, 1.25]
P= 0.5
Baseline to Day 29
HR: 0.98 [0.87, 1.10]
P= 0.7
Baseline to Day 63
HR: 1.02 [0.79, 1.33]
P= 0.9
PFS
OS
log2(Hazard) for 75th–25th difference in CM9
e
Baseline to Day 29
HR: 1.11 [1.00, 1.24]
P= 0.05
Baseline to Day 43
HR: 1.12 [0.91, 1.38]
P= 0.27
Baseline to Day 29
HR: 1.23 [1.03, 1.46]
P= 0.02
Baseline to Day 43
HR: 1.14 [0.88, 1.47]
P= 0.31
log2(Hazard) for 75th–25th difference in CM38

Figure S6. Association between change in sPD-L1 and gene expression in CheckMate 009/RCC and CheckMate 038-P1/Melanoma

Data shown are for patients from CheckMate 009 (a,b,d,e) and CheckMate 038-P1(c). Three outliers for change in sPDL1 level at day 29 were removed from CheckMate 009 dataset because they skewed the correlation analyses. BOR is indicated by gold (PD), blue (SD), aqua (CR or PR) or grey (ND).

a. CheckMate 009 patients are ordered by their change in sPDL1 level at day 29, shown in the barchart. Vertical line indicates the median change in sPDL1 level at day 29, calculated on the entire patient cohort. Bars are colored by the change in Neutrophil count for a day 29 versus a baseline biopsy from the same patient. Samples with a Neutrophil count of zero at both timepoints were omitted. Scale for score is -0.25 to 0.5 (blue to red).
b. CheckMate 009 patients are ordered by their change in sPDL1 level at day 29, shown in the barchart. Vertical line indicates the median change in sPDL1 level at day 29, calculated on the entire patient cohort. Bars are colored by the level of activated NK cells, determined by Cibersort, in a baseline biopsy from the same patient. Scale for cell content is 0 to 0.19 (white to black).
c. CheckMate 038 patients are ordered by their change in sPDL1 level at day 29, shown in the barchart. Vertical line indicates the median change in sPDL1 level at day 29, calculated on the entire patient cohort. Bars are colored by the level of activated NK cells, determined by Cibersort, in a baseline biopsy from the same patient. Scale for cell content is 0 to 0.8 (white to black).
d. Change in sPDL1 level at day 29 in CheckMate 009 patients. Data are grouped by predicted ccrcc subtype, predicted from the baseline biopsy: ccrcc1 (n = 18), ccrcc2 (n = 12), ccrcc3 (n = 3), ccrcc4 (n=14). *P* values for distribution of BOR categories are from the Kruskal-Wallis rank sum test.
e. Rate of refractory patients (best response of PD in CheckMate 009) in groups based on partition at the median change of sPDL1 at Day 29 (180pg/ml). Data is presented for patients of the ccrcc1 subtype (n = 18) and the ccrcc4 subtype (n = 14). *p*-values from Fisher exact test.

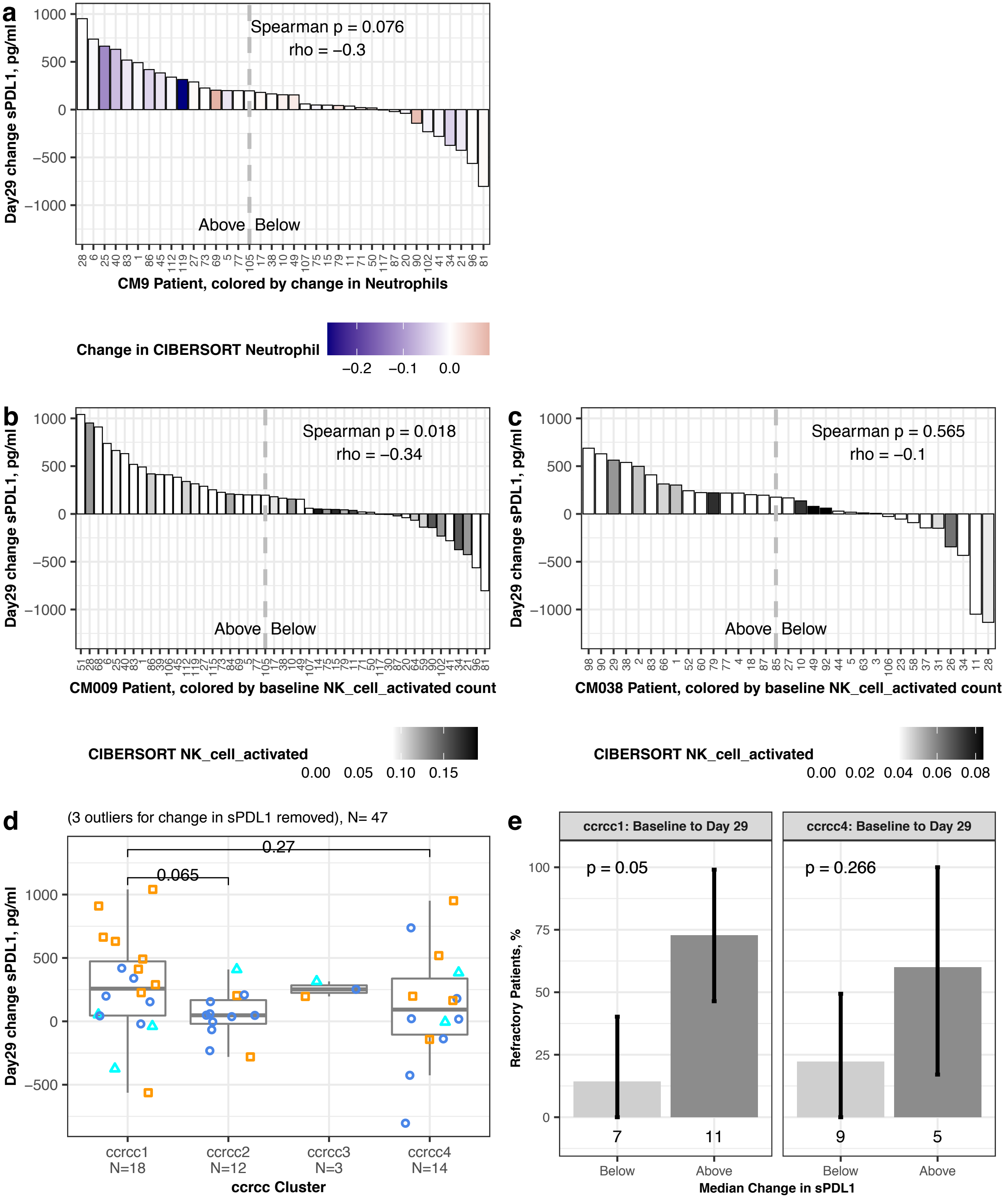
a
Spearman p = 0.076
rho = −0.3
Above
Below
Day29 change sPDL1, pg/ml
CM9 Patient, colored by change in Neutrophils
Change in CIBERSORT Neutrophil
−0.2
−0.1
0.0
b
Spearman p = 0.018
rho = −0.34
Above
Below
Day29 change sPDL1, pg/ml
CM009 Patient, colored by baseline NK_cell_activated count
CIBERSORT NK_cell_activated
0.00
0.05
0.10
0.15
c
Spearman p = 0.565
rho = −0.1
Above
Below
Day29 change sPDL1, pg/ml
CM038 Patient, colored by baseline NK_cell_activated count
CIBERSORT NK_cell_activated
0.00
0.02
0.04
0.06
0.08
d
(3 outliers for change in sPDL1 removed), N= 47
0.27
0.065
Day29 change sPDL1, pg/ml
ccrcc1 N=18
ccrcc2 N=12
ccrcc3 N=3
ccrcc4 N=14
ccrcc Cluster
e
ccrcc1: Baseline to Day 29
ccrcc4: Baseline to Day 29
p = 0.05
p = 0.266
Refractory Patients, %
7
11
9
5
Below
Above
Below
Above
Median Change in sPDL1
